# A global comparison of building decarbonization scenarios by 2050 towards 1.5–2 °C targets

**DOI:** 10.1038/s41467-022-29890-5

**Published:** 2022-06-02

**Authors:** Clara Camarasa, Érika Mata, Juan Pablo Jiménez Navarro, Janet Reyna, Paula Bezerra, Gerd Brantes Angelkorte, Wei Feng, Faidra Filippidou, Sebastian Forthuber, Chioke Harris, Nina Holck Sandberg, Sotiria Ignatiadou, Lukas Kranzl, Jared Langevin, Xu Liu, Andreas Müller, Rafael Soria, Daniel Villamar, Gabriela Prata Dias, Joel Wanemark, Katarina Yaramenka

**Affiliations:** 1 UNEP-DTU Partnership, Marmorvej 51, 2100 Copenhagen, Denmark; 2 IVL Swedish Environmental Research Institute, Aschebergsgatan 44, 411 33 Gothenburg, Sweden; 3 European Commission, Joint Research Centre (JRC), Westerduinweg 3, 1755 LE Petten, The Netherlands; 4 National Renewable Energy Laboratory (NREL), 15013 Denver W Pkwy, Golden, CO 80401 USA; 5 Universidade Federal do Rio de Janeiro, Av. Pedro Calmon 550, Rio de Janeiro, 21941-901 Brazil; 6 Lawrence Berkeley National Laboratory (LBNL), Cyclotron Rd, Berkeley, CA 94720 USA; 7 TU Wien, Energy Economics Group, Gußhausstraße 25, 1040 Vienna, Austria; 8 SINTEF Community, Høgskoleringen, 7B 7034 Trondheim, Norway; 9 National School of Development, Peking University, 5 Yiheyuan Rd, Haidian District, 100871 Beijing, China; 10 Universidad San Francisco de Quito, Diego de Robles y Vía Interoceánica, Department of Mechanical Engineering. Campus Cumbayá, 170901 Quito, Ecuador; 11 Escuela Politécnica Nacional (EPN), Departamento de Ingeniería Mecánica, Av. Ladrón de Guevara 253, Quito, Ecuador

**Keywords:** Climate sciences, Energy and society

## Abstract

Buildings play a key role in the transition to a low-carbon-energy system and in achieving Paris Agreement climate targets. Analyzing potential scenarios for building decarbonization in different socioeconomic contexts is a crucial step to develop national and transnational roadmaps to achieve global emission reduction targets. This study integrates building stock energy models for 32 countries across four continents to create carbon emission mitigation reference scenarios and decarbonization scenarios by 2050, covering 60% of today’s global building emissions. These decarbonization pathways are compared to those from global models. Results demonstrate that reference scenarios are in all countries insufficient to achieve substantial decarbonization and lead, in some regions, to significant increases, i.e., China and South America. Decarbonization scenarios lead to substantial carbon reductions within the range projected in the 2 °C scenario but are still insufficient to achieve the decarbonization goals under the 1.5 °C scenario.

## Abstract (web summary)

Building decarbonization has an important role to play in achieving global emissions reductions targets. Here the authors find that stated policy scenarios are insufficient to achieve building decarbonization goals globally, while ambitious decarbonization scenarios are still not sufficient to achieve goals under the 1.5 °C scenario.

## Introduction

Buildings make up around one-third of total final energy consumption (FEC) and CO_2_ emissions globally in 2020^1^, and they will likely play a critical role in the global low-carbon transition. However, without further climate policy, the energy used in buildings can increase by 46–73% in 2050 from 128 EJ in 2019, driven by population growth, greater diffusion and use of energy-consuming devices, and increasing living standards in developing countries. In particular, the baseline scenarios of the five “shared socioeconomic pathways” (SSPs) show a paradigm shift in the energy demand of buildings: the demand of appliances, lighting and space cooling increases, whereas that of space heating and cooking declines over the next three decades. Furthermore, these scenarios show that the importance of developing countries to GHG mitigation strategies increases, and electricity becomes the main energy carrier globally^2–4^. Meanwhile, to restrict the increase of global temperature to less than 1.5 °C–2 °C, we need CO_2_ emissions reductions within the range of 50–60%^5^ to 90%^6^ in 2050, depending on the scenario and model^5–8^. These reductions can be realized even with increases in energy demand (changes between −3% and +50% in energy demand are foreseen depending on the decarbonization scenario). To achieve these targets, the building sector will need to employ a suite of strategies including new construction of net-zero carbon buildings, high rates of energy renovation in existing buildings, low-energy-consumption behavioral practices, development of new low-energy building technologies and appliances, deployment of centralized and decentralized renewable energy sources (RESs), and widespread electrification of building technologies^6,8–12^. The design of these strategies at scale will require the development of national and transnational roadmaps based on feasible decarbonization scenarios^10,11^. Existing global models^5–10^ can be valuable for identifying key paths as they encompass major socioeconomic trends, but they are frequently limited by their spatial and temporal scopes and resolutions and levels of technology details. On the other hand, sector-specific models, though lacking some of the socioeconomic indicators in global models, can provide a more detailed understanding of both the current conditions and possible future evolution of building energy demand patterns and insights into technology trends, implementation costs, and resulting energy use^13,14^.

Against this background, our study aims to share insights from national building sector models to describe carbon mitigation scenarios by 2050, including descriptions of factors such as energy, CO_2_ emissions, and associated socioeconomic indicators, and compare them to results from global models in line with 1.5 °C–2 °C scenario goals that provide information on mitigation measures on sub-sectoral levels. To this end, we compiled results from 32 countries across four continents, namely, Asia, Europe, and South and North Americas—a sample that we consider sufficiently representative of the building stock worldwide as it together covers more than 60% of global building emissions while accounting for most of the world regions and building typologies according to energy structure and development^15,16^. To the best of our knowledge, this is the most comprehensive transnational compilation of building carbon mitigation bottom-up sectoral studies, and it provides a strong, data-informed foundation for developing coordinated international building decarbonization plans. The large array of sector-specific models used in this study to examine decarbonization pathways for building is, therefore, of potentially great value to understanding the technologies and efficiency measures, including the interactions between them. More importantly, the level of aggregation also allows for comparison of key decarbonization indicators between national sectoral and global modeling.

In the compilation of sectoral and national modeling studies, we identify two scenarios for each country: a reference scenario (RS), which assumes the energy and climate policies currently enacted along with moderate, expected policy enhancements, and various decarbonization scenarios (DSs), which represent the most ambitious potential actions based on techno-economic and policy feasibility within each national context. The DSs include the combined efforts for energy saving, efficiency, and decarbonization of both energy demand and supply. The specific measures depend on each country’s framework conditions.

Table 1 presents an overview of the models used in this study, including model classification^13^ and spatial and temporal resolution for the regions and countries covered. The detailed assumptions of each model and respective scenarios for the drivers behind technology and demand development (i.e., policy instruments, energy price, demographic and floor area development, demand drivers, primary model inputs, and accounting of RES) are provided in Supplementary Table 1 and 2.

**Table 1:** Model overview per region and country. Subsectors: R, Residential; C, Commercial.

Continent/Region/Country [Subsector]	Model	Classification: Quadrant* according to ref. ^13^	Spatiotemporal resolution**	Scenarios (Reference scenario)	Reference
**Europe**					
Northern and Western (NW Europe)					
Sweden (SWE) [R, C], Germany (DEU) [R], France (FRA) [R, C], United Kingdom (GBR) [R, C]	ECCABS	Q4 (Bottom- up/White- box)	Hourly energy demand, annual investment decisions, national climate zones	BAU-TE BAU-T	^33^
Norway (NOR) [R, C]	RE-BUILDS	Hybrid: Q1/Q2/Q4 (technological, system dynamics and physics simulation)	Annual, national scale	Baseline Ambitious zero-emission building scenario	^30^
Austria (AUT) [R, C], Belgium (BEL) [R, C], Denmark (DNK) [R, C], Estonia (EST) [R, C], Finland (FIN) [R, C], France (FRA) [R, C], Germany (DEU) [R, C], Ireland (IRL) [R, C], Latvia (LVA) [R, C], Lithuania (LTU) [R, C], Luxembourg (LUX) [R, C], The Netherlands (NLD) [R, C], Norway (NOR) [R, C], Sweden, (SWE) [R, C], United Kingdom (GRB) [R, C]	Invert/EE-lab	Q4 (Bottom- up/White- box)	Monthly, national scale	Reference Diversification Directed vision Localization National_ champions	^34^
Germany (DEU) [R]	CoreBee	Q4 (Bottom- up/White- box)	Annual energy demand and consumption, national scale	1 2 3	^35^
			Annualized investment costs (50—year building lifetime)		
Southern and Eastern (SE Europe)					
Greece (GRC) [R]	CoreBee	Q4 (Bottom- up/White- box)	Annual energy demand and consumption, national scale	1 2 3	^35^
Spain (ESP) [R, C]	ECCABS	Q4 (Bottom- up/White- box)	Hourly energy demand, annual investment decisions, national climate zones	BAU-TE BAU-T	^33^
Bulgaria (BGR) [R, C], Cyprus (CYP) [R, C], Croatia (HRV) [R, C], Greece (GRC) [R, C], Italy (ITA) [R, C], Malta (MLT) [R, C], Portugal (PRT) [R, C], Slovenia (SVN) [R, C], Spain (ESP) [R, C], Czech Republic (CZE) [R, C], Hungary (HUN) [R, C], Poland (POL) [R, C], Portugal (PRT) [R, C] Romania (ROU) [R, C], Slovakia (SVK) [R, C],	Invert/EE-lab	Q4 (Bottom- up/White- box)	Monthly, national scale	Reference Diversification Directed_vision Localization National_champions	^34^
North America					
United States of America					
United States of America (USA) [R, C]	Scout	Hybrid Q1/Q4 (technological-econometric + end-use distribution)	Annual, AIA (American Institute of Architects) Climate Zone	AEO2019-SDS AEO2019-Ref AEO2019-HR	^36^
South America and Caribbean					
South America (SA)					
Brazil (BRA) [R, C]	BLUES v2.0	Multiple Quadrants (Hybrid)	Annual, five Brazilian macro-regions	adb ssp1_bau ssp1_pol ssp3_bau ssp3_pol ssp4_bau ssp4_pol ssp5_bau ssp5_pol	^37^
Ecuador (ECU) [R, C]	ELENA	Q2 (Top-down/ White-box)	12-month resolution, with a typical day divided into five time periods	RS DDP	^38^
			4 regions		
Asia					
Eastern Asia (excluding Japan)					
China (CHN) [R, C]	DREAM	Q4 (Bottom- up/White- box)	Annual, Chinese climate zone division	Reference Techno-economic-potential (TEP) Electrification plus efficiency plus clean transformation (Electrification)	^39^

*Q1 (Top-down/ Black-box) estimates aggregate building energy use from sector-wide socioeconomic and/or technological variables. Q2 (Top-down/ White-box) represents physical causality at the aggregate building and technology stock level. Q3 (Bottom- up/Black- box) attributes building-level energy use to particular energy end uses (e.g., space heating, hot water usage, and household appliances) based on statistical analysis of historical data. Q4 (Bottom- up/White- box) simulates the physical relationships of processes at the building or energy end-use level. Multiple quadrants (Hybrid) combine elements of the modeling approaches across the four classification quadrants.

** Each model makes specific assumptions for the calculation of the energy requirements. This includes decisions on the level of resolution, geographical scope or set-point temperatures among others.

This work integrates building stock energy models for 32 countries across four continents to create carbon emission mitigation reference scenarios and decarbonization scenarios by 2050, covering 60% of today’s global building emissions. These decarbonization pathways are compared to those from global models. Results demonstrate that reference scenarios are in all countries insufficient to achieve substantial decarbonization and lead, in some regions, to significant increases, i.e., China and South America. Decarbonization scenarios lead to substantial carbon reductions within the range projected in the 2 °C scenario but are still insufficient to achieve the decarbonization goals under the 1.5 °C scenario.

## Results

### Status quo: socioeconomic, climate, and energy indicators

**Fig. 1:**
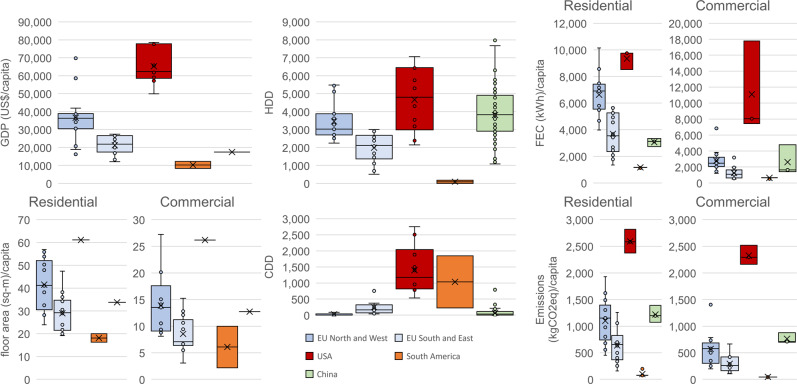
Status quo (Year 2020): median of the gross domestic product (GDP), floor area, heating degree days (HDD) and cooling degree days (CDD), final energy consumption (FEC), and CO_2_ emissions by the studied region (Northern and Western (NW) Europe and Southern and Eastern (SE) Europe, and South America) or country (the USA and China). In this plot, each region is described by the countries within the region. In the case of the USA and China, as they are the only countries within their region modeled in this study, their distributions are taken as the distributions of their administrative divisions.

National socioeconomic contexts and climate heavily affect the energy demand and resulting carbon emissions from buildings^17^. Figure 1 shows that in 2020 the gross domestic product (GDP) is related to the floor area, energy demand, and CO_2_ emissions of a region. The USA has the highest GDP per capita (65 kUS$/capita) and FEC (~10,000 kWh/capita), followed by Northern and Western (NW) European countries (36 kUS$/capita and ~5,000 kWh/capita). CO_2_ emissions per capita are, on average, higher in residential buildings than in commercial buildings in all regions; however, this is not so for FEC. This means that per capita the residential sector is more carbon intensive than the commercial sector. Similarly, floor area is higher for residential buildings than for commercial buildings in all countries except the USA and China. Among the climatic indicators, heating degree days (HDD) are much higher to cooling degree days (CDD) across countries compared. As a result, HDD show a strong correlation with FEC. Note that the impact of future climate change on building energy demand is not considered in any of these scenarios, though related models were previously used to investigate the effects of climate change in buildings energy consumption for the USA^18^ and Sweden^19^.

Considering individual countries in these regions, Sweden (NW Europe) has the most heating-intensive climate with 5,225 HDD and almost no CDD, whereas Ecuador (in South America) has the lowest heating intensity with no HDD and 224 CDD. The USA is climatically diverse with regions of both high HDD and CDD, but considering the population-weighted average, the USA has the largest cooling need in this study at nearly 1,200 CDD. The countries with the highest GDPs are Luxembourg (NW Europe) and the USA (69 kUS$/capita and 65 kUS$/capita, respectively). In terms of regions, the floor area follows a similar trend, with the highest value in the USA (R: 61 m^2^/capita, C: 26 m^2^/capita) and the lowest in Ecuador (R: 16 m^2^/capita, C: 2 m^2^/capita). The CO_2_ intensity of energy demand also varies significantly across countries (see Supplementary Table 3.1 and Supplementary Table 3.2), depending on both the energy carrier mix and the CO_2_ intensity of electricity and heat generation. In 2020, China had the highest CO_2_ intensity of FEC at 0.48 kg CO_2_/kWh, followed by the USA at 0.30 kg CO_2_/kWh, Europe at 0.20 kg CO_2_/kWh, and finally South America at 0.11 kg CO_2_/kWh, which can be explained by the abundancy of hydroelectric plants. As a consequence of these trends, the USA had the highest CO_2_ emissions rate (~2.5 t CO_2_/capita), followed by China (~1.0 t CO_2_/capita), NW Europe and Southern and Eastern (SE) Europe (<0.5 t CO_2_/capita), and South America (~0.1 t CO_2_/capita). Thus, it is clear that effective decarbonization strategies will differ with regions. For instance, the expected growth of the Brazilian building stock can lead to an increase in the energy demand. This, in turn, offers the possibility of implementing adequate urban design and roadmaps toward 2050. In regions with higher GDP and floor area per capita, such as the USA or NW Europe, the focus in the upcoming years is expected to be on the adoption of energy efficiency measures and decarbonization of the energy supply, among others. Thus, the socioeconomic, climate, and energy indicators considered in this work have a strong impact on the definition of scenarios and decarbonization pathways.

### Reference and decarbonization scenarios: Ambitious enough to achieve 1.5 and 2 °C targets?

**Fig. 2:**
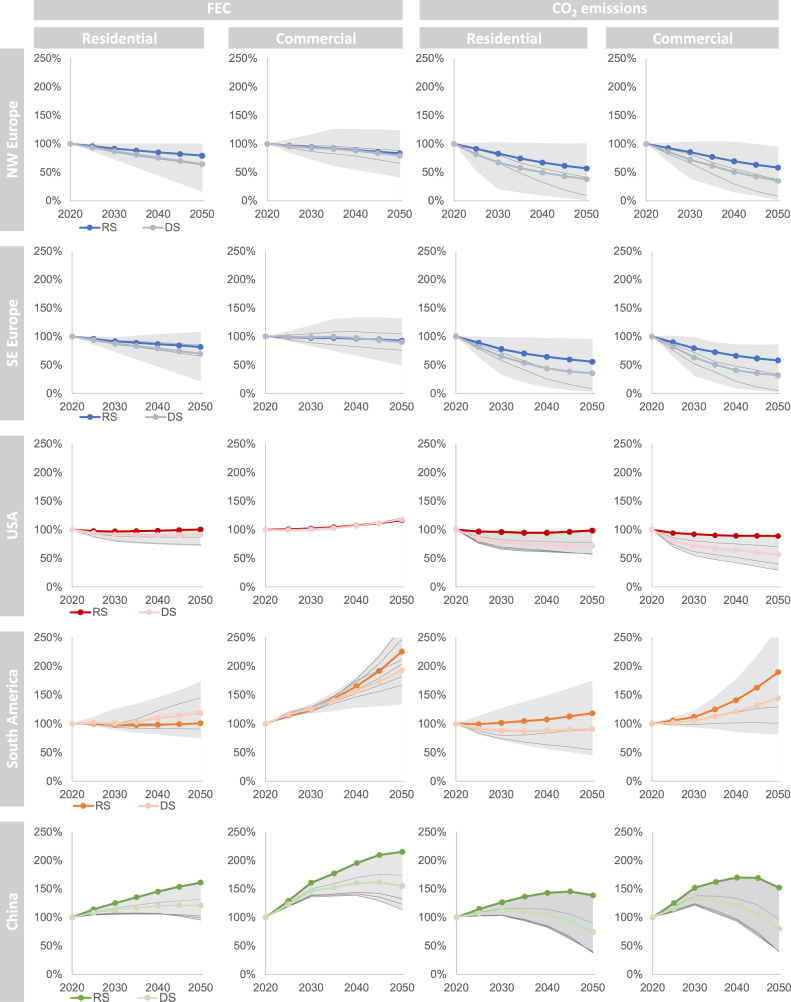
Annual FEC and CO_2_ emissions in residential and commercial subsectors region-wise (NW and SE Europe, South America) and country-wise (USA and China) in the reference scenario (RS) and decarbonization scenarios (DSs) until 2050, shown as percentages of the corresponding value in 2020. The gray area shows the range of values between the minimum and maximum from both the RS and DSs across the regions.

For each study region we ran the individual sectoral models and scenarios listed in Table 1. The resulting FEC and CO_2_ emissions towards 2050 are presented in Fig. 2. A more comprehensive and granular description of the mitigation actions in these models are presented in Supplementary Table 2. Additionally, a high-level comparison of key components of the decarbonization strategies (floor area, renovation rates, share of electricity in total FEC, electricity from RES, carbon intensity of electricity, and total emissions) between sectoral and global models is summarized in Supplementary References 1. In the subsections below, we discuss region-wise the scenario ambitions, in terms of FEC and CO_2_ emissions, and the required measures to achieve them for various national contexts. This is followed by a comparison of CO_2_ emission reduction between the global aggregation of DS from sectoral models and the 1.5 °C–2 °C scenarios of global models.

In NW Europe, the FEC and CO_2_ emissions are similar across the residential and commercial sectors. The four DSs conducted with Invert/EE-Lab yield FEC reductions in the range of 29%–32% in 2050 compared to 2020. The highest reduction is seen in the scenarios Directed Vision and National Champions. The former focuses on energy efficiency and district heating policies, with emissions reductions of 89% between 2020 and 2050. The latter assumes differentiated country-specific policies to meet individual national targets and leads to a 70% reduction. In this second scenario, we see considerable shares of natural gas for heat remaining in some countries, with the open question whether, and at what cost, this could be supplied by green gas. Further, in the two scenarios Diversification, which includes a highly diversified heating technology stock, and Localization, which focuses on local resources, the FEC decreases by roughly 30% in both scenarios. The resulting emission reductions are similar to the reduction in Directed Vision. Technology wise, Diversification and Localization include greater deployment of heat pumps and direct use of biomass than Directed Vision. An additional fifth DS was modeled with ECCABS (BAU-T) for three countries (the United Kingdom, Sweden, and France), and it yielded in FEC and CO_2_ emissions reductions of 13%–52% and 47%–76%, respectively, in 2050 compared to the 2020 values. In the results of the ECCABS model, the energy reduction potentials for Sweden and the United Kingdom are substantially higher than the estimates from Invert/EE model but the reduction potential is lower for France. Similar to the energy reductions, ECCABS estimates higher reductions of CO_2_ emissions compared to Invert/EE because of the comparatively increased decarbonization of the energy supply and large rollout of heat pumps. A separate scenario modeled in CoreBee for the residential stock of Germany estimates that an annual 3% refurbishment rate of building envelopes and heating/cooling system upgrades combined with small-scale RES, and prosumer strategies for micro combined heat and power (3%_RR), can reduce FEC by 85% and emissions by 89% in 2050, compared with the RS. The Norwegian DS modeled in RE-BUILDS, results in a 56% energy demand reduction compared to 2020, assuming a large-scale rapid introduction of zero-emission building technologies in new and renovated buildings. The absolute savings in the DS vary largely from 0.7 to 16 Mt CO_2_ and are generally half of the RS. About 70% of the emissions saving potential comes from residential buildings including the decarbonization of the power sector. The differences in the model results are driven by the specific techno-economic and policy assumptions (as shown in Supplementary Table 2.1).

For SE Europe, the results from the Invert/EE-lab model show reductions of 27%–31% in FEC in 2050 compared to 2020. Using the same scenarios as those for NW Europe, the highest CO_2_ emission reductions are achieved in Directed Vision, with a decrease of 90%. In Diversification and Localization, the FEC decreases by 27% owing to a strong uptake of heat pumps, photovoltaics, solar thermal systems, and biomass. National Champions leads to reductions of 27% in FEC and 85% in CO_2_ emissions. As in the case of NW Europe, this scenario has the highest remaining share of natural gas in 2050. Compared to NW Europe, SE Europe shows considerable electricity demand for space cooling because of the warmer regional climate. All scenarios for NW and SE Europe presume a strong decarbonization of the electricity-based and district heating supply systems. ECCABS-DS shows that the FEC of buildings in Spain reduce by 53% in 2050 compared to 2020, with a corresponding decrease of 59% in emissions. The resulting energy use from these scenarios is substantially lower than the more conservative estimates of ~20% reductions from the Invert/EE-lab model. Nonetheless, the resulting emission reductions are lower in the Invert/EE-lab model. This is partially attributed to the fact that Invert/EE-Lab model adopts more conservative policy assumptions that affect the cost-effectiveness of technology options and renovation measures, while simultaneously assuming a more radical decarbonization of the energy system. Results from CoreBee show that the residential sector in Greece (3% RR scenario) can reduce its FEC by 79% and CO_2_ emissions by 88% in 2050, compared with the RS.

In the USA, the population growth between 2020 and 2050 is expected to be 17%. This population growth will drive baseline increases in energy demand. In a DS with aggressive deployment of building efficiency measures, incentivized electrification of building technologies, and a high share of renewable electricity generation (AEO2019-SDS), the building FEC can be reduced by 21% in 2050 with associated emission reductions of 1,018 Mt CO_2_. Total emissions under this scenario are 64% lower than those in 2020, while the modeled RS (AEO2019-Ref) has reductions of just 6% compared to 2020. A second DS assumes only higher renewable electricity supply without further demand reductions (AEO2019-HR), and this scenario achieves reductions by 53% in 2050, indicating the strong influence of electricity supply decarbonization on potential emissions reductions from buildings in the USA. There are minimal demand reductions in the commercial sector compared to the reductions in the residential sector, partially due to the lower floor area in the commercial sector. Of the avoided emissions in AEO2019-SDS that can be directly attributed to reductions in building demand, the greatest contribution comes from residential building efficiency measures (91% in 2050)—including the deployment of heat pumps for residential space and water heating, improved building controls, highly insulating thermal envelopes, and air-sealing technologies.

South America shows an increase in FEC and CO_2_ emissions in both the residential and commercial sectors—the latter presents the steepest increase across all studied regions. In Brazil, the energy demand is expected to increase annually by 0.9% between 2020 and 2050 because of a higher demand from commercial activities, which are an effect of economic development. However, in a scenario assuming an increase in energy efficiency and electrification of building technologies (SSP4_pol), FEC and electricity consumption will fall by 5% and 42%, respectively, in 2050, with 82% of the avoided emissions originating from residential buildings. This reduction is achieved by considerable deployment of energy efficient residential technologies (e.g., more efficient lighting technologies and electrical appliances such as refrigerators, water heaters, air conditioning, and fans), combined with a shift from traditional fuels (e.g., firewood and coal) to natural gas and liquefied petroleum gas (LPG). In Ecuador, the FEC in the commercial sector is the same for both the RS and DS, whereas that in the residential sector varies in each scenario. FEC in the DS is lower than that in the RS for most of the final energy uses, except cooking (which is constant) and electric appliances (which significantly increases). In 2050, the total FEC is ~60 TWh for both scenarios, but the emissions for 2050 in the DS are 6% lower than those modeled in the RS (8 and 9 Mt CO_2_, respectively). The RES for electricity generation is expected to reach a share of 95% in 2050 in the DS. Additionally, two specific ambitious strategies can further support the decarbonization of the Ecuadorian building sector beyond what was included in the DS. First, cooking can be electrified to a greater extent by replacing LPG stoves by induction stoves. Second, increased shares of old appliances can be replaced with new and more efficient equipment.

In the Chinese RS, economic growth drives the increase in energy demand and CO_2_ emissions in both residential and commercial buildings. Heating and cooling demands also increase with economic growth. The cooling demand per unit floor area in China’s northern climate zones rise from 5–7 kWh/m^2^ in 2020 to 4–19 kWh/m^2^ in 2050. In the transition climate zones, the heating demands per unit floor area evolve from ~25 kWh/m^2^ to 10–68 kWh/m^2^ over the same time period. The DS (High-efficiency) assumes an increasing market penetration of net-zero energy buildings (NZEB), large-scale deployment of high-efficiency appliances, retrofits in the existing buildings (particularly in northern China), and a significant uptake of distributed RES. Furthermore, in the DS, the CO_2_ emissions intensity of electricity production is reduced from 0.54 kg CO_2_/kWh in 2020 to 0.09 kg CO_2_/kWh in 2050, with the share of carbon-free generation sources increasing from 41% in 2020 to 89% in 2050. To leverage the lower-emission electricity from the power grid, an additional DS building electrification scenario was modeled. This scenario reflects a replacement of 50% of coal and natural gas equipment by heat pumps by 2050 for space heating needs in urban commercial and residential buildings in northern China. As a result, 70% of FEC is met by electricity in 2050 (~80% for commercial buildings and ~62% for residential buildings).

By adding up regional results, RS leads to a global 18% CO_2_ emissions increase — from 5.7 GtCO_2_ in 2020 up to 6.7 GtCO_2_ in 2050. This is mainly driven by China, responsible of 70% of this increase. Per region, EU (both NW and SE) and the USA show reductions of 42% and 6% respectively, while China and SA show increases of 44% and 32%. Conversely, DS leads to emission decreases of around two thirds of current levels by 2050 down to 1.9 GtCO_2_. This means that the level of ambition of the national developments represented in the DSs would achieve the 2 °C scenario goals, yet appear insufficient to maintain global temperature below 1.5 °C. In the conclusions, based on the data of Supplementary Notes/Methods 1, we elaborate on the additional efforts that would be required to achieve the 1.5 °C scenario goals.

### Socioeconomic insights from the DSs

**Fig. 3:**
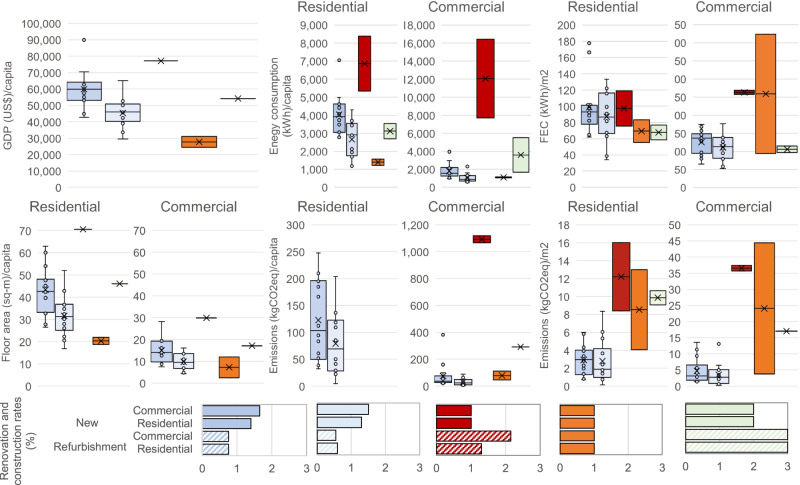
Decarbonisation Scenarios (Year 2050). Gross domestic product (GDP), floor area, annual total final energy consumption (FEC) and CO_2_ emissions per capita and per m^2^ across regions and countries.

Each of the DS is built on the distinct socioeconomic and climatic conditions of each country, resulting in different FEC per capita by 2050 (Fig. 3). Of the five regions, the USA continues to have the highest relative FEC in 2050 at ~8,100 kWh/cap, followed by NW Europe and China. Although the USA has the highest total FEC value, the reduction compared to the RS is still substantial (~15%). NW and SE Europe have the greatest relative FEC reductions at 40%, with NW Europe finishing at ~3,000 kWh/capita in 2050 and SE Europe at ~1,500 kWh/capita. The floor area varies substantially across regions but is approximately the same in both the DS and RS. In both cases, the USA has the highest floor area (~100 m^2^/cap) of which nearly 70% is in the residential sector; furthermore, 75% of the 2050 modeled floor area already exists in 2020.

In 2050, China has the second highest average FEC at ~4000 kWh/capita, which represents a 25% increase from the FEC in 2020, mostly because of a socioeconomic growth — characterized by a rapid urbanization (from 61% of the population living in urban areas in 2020 to 80% in 2050) —, and standards of living; thus, a larger diffusion and use of appliances. The FEC increase is mainly due to a rise in the floor area per capita in the residential buildings. In developing economies, increased living standards are expected by 2050. In South America, the DSs have an average annual FEC of ~1,000 kWh/cap by 2050: an increased demand for energy services is offset by the electrification of most building energy uses and the adoption of efficient technologies. In Brazil, the FEC in the DS does not increase remarkably in most scenarios for the residential sector. In Ecuador, by 2050, the population is expected to increase by nearly 45%, while the average household size is expected to fall from 3.8 members to 2.7, thereby doubling the number of households. However, new construction homes are expected to adopt the electrification of water heating and cooking, thereby reducing onsite GHG emissions from LPG combustion and firewood stoves. In parallel, the demand for electric appliances is predicted to double because of an increased uptake of smart appliances.

**Fig. 4:**
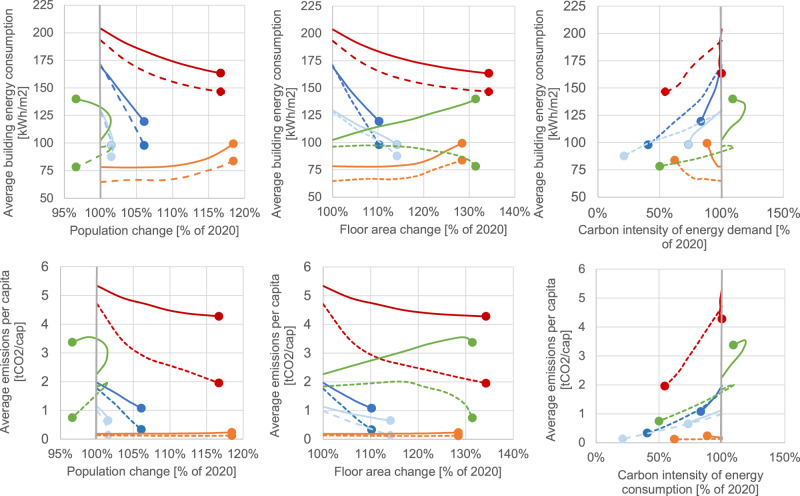
Changes in demands and services from 2020 to 2050 (X-axes: population, floor area, and carbon intensity of final energy consumption (FEC) change. Y-axes: average building energy demand and CO_2_ emissions per capita in the different scenarios by regions and countries). The oval arrow shows the direction of evolution over time, and the oval indicates the value for 2050.

Figure 4 shows the relative evolution (with 2020 as the reference year) of both socioeconomic and energy variables. The comparison of the relative evolution across regions provides valuable insights. First, graphs (i-iii, y-axis: average building consumption per floor area) show that in developed regions, namely, the USA and NW and SE Europe, energy consumption per floor area decreases in both scenarios (the RS and DSs). The opposite is observed for South America, with energy consumption increasing per unit floor area, while in China, it depends on the scenario: FEC increases in the RS and decreases in the DSs. From the population and floor area relationships (represented on the x-axes of charts i and ii, respectively), South America shows the largest population growth (18%), while the USA has the largest floor area growth (34%), partially because of its 17% population growth. Europe has a modest population growth of 6%, whereas the population in China decreases by 3%. In all regions, the ratio of floor area per capita increases remarkably, especially in China. Particularly interesting is the comparison between FEC and carbon intensity (chart iii), which shows that energy consumption plunges more rapidly than carbon intensity in developed regions (the USA and NW/SE Europe) in the RS, while the DSs are driven by large carbon intensity reductions and moderate efficiency improvements. In South America, there are steep increases in energy use but modest improvements in carbon intensity. With regard to China, the RS and DS yield drastically diverging cases and energy and carbon intensity relationships. In terms of the average emissions per capita (charts iv-vi), although South America has the largest population change (19%), its emissions remain consistently the lowest along with those of SE European countries, in terms of both per population and floor area change (~0.1 tCO_2_/cap). In the USA, which has the second largest population growth (17%), CO_2_ emissions per capita decreases to 2 tCO_2_/cap in the DS. The carbon intensity of FEC (chart vi), decreases in all the DS of the five studied regions.

A comparison of the results shown in Fig. 4 to the socioeconomic 2050 conditions shown in Fig. 3 clearly shows the interdependent relationships of GDP, population, floor area, FEC, and emissions. The floor area per capita increases among the developing economies (China, Brazil and Ecuador), while in developed economies, it remains consistently high.

Our assumptions are generally aligned with the global models in that these models assume increases of 32% (to 29 m^2^/cap) and 50% (to 9 m^2^/cap) for residential and commercial buildings, respectively, for the global south, or that half of the floor area additions will be in sub-Saharan Africa and India^7,20^. Our assumptions for developed economies and conclusions are aligned with the observed trends of declining household sizes^21–24^ but are in contrast with the embedded assumptions of maintaining or reducing floor areas per capita in many global models^1,6,7^ (see Supplementary Notes/Methods 1). The assumptions vary in the global models: in the global north, the density of residential buildings is roughly constant, whereas that of commercial premises increases by 44% to 23 m^2^/cap. Worldwide, estimates from the global models range from absolute (not per capita) increases of more than twofold by 2070^20^ to constant trends converging to 30 m^2^/cap in 2050^7^, with other studies assuming no changes from the baseline scenario^6^. Recent studies suggest that 10–15 m^2^/capita can provide decent living standards globally with minimum energy demand^23,24^. However, a remaining question is whether developed countries that maintain most of their building stock into the future will transition to a more densified living arrangement.

## Discussion

This study clarifies potential decarbonization pathways for the building sector towards 2050 climate targets by comparing national sectoral models results with global models. The contribution of this work is to: (1) provide detailed analysis of decarbonization scenarios based on measures defined by national contexts; (2) offer a high level of granularity and disaggregation of the results based on an empirical characterization of the stock defined by buildings’ physical properties (e.g., size, age, thermal properties, etc.); and (3) compare and discuss the results with those from global models in terms of total FEC and CO_2_ emission savings. Such a detailed analysis had not been done before using building stock bottom-up sectoral models covering world regions totaling 60% of the global sector CO_2_ emissions.

Our study presents an analysis of the RS and DSs in buildings through 2050 across 32 countries based on national building sector models. The RS scenarios, which assume the progression of current policy and techno-economic frameworks, show approximately 1 GtCO_2_ emission increase by 2050 globally. The DSs, which built upon the most ambitious techno-economic and policy pathways within each national context, result on average renovation rates by 1.4%, an average share of direct RES of 38%, and electrification of FEC by 38–80%. These attain a total reduction of almost 4 GtCO_2_ emissions by 2050 within the range projected in the 2 °C degrees scenario but still insufficient to achieve the decarbonization goals for the 1.5 °C degrees scenario^9^.

To align with 1.5 °C scenarios, the national decarbonization strategies are required to increase current annual renovation rates to a total of 2.4%, and to increase the share of electrification of buildings’ FEC by 4–14% to an average of around 50% in all regions. Furthermore, by increasing the share of direct RES in the energy mix by 3–30% to a global share of around 70%, the carbon intensity of electricity production should be 4–6 times lower than that assumed in the DSs (at least below 40 gCO_2_/kWh globally). To this aim, only a comprehensive set of sufficiency, efficiency, and RES actions in the building sector allows to achieve the most ambitious climate goals.

Our analysis concludes that the aggregation of national models complements assessments made by global models in the building sector. Further, it provides a more detailed overview based on the incorporation of specific national/regional contexts, including building stock characteristics and socioeconomic trends. Therefore, there is a great potential for national models to be tailored to government decisions addressing context-specific challenges and, ultimately, contributing to build evidence towards global climate goals.

## Methods

### Study design

We build on the outputs of published national bottom-up building sectoral modeling studies. These models assess the energy demand and carbon emissions of building stocks and develop pathways and scenarios for energy and carbon emission reduction. Unlike global models, the assessment of the scenarios is done through the characterization of building stocks defined by buildings’ physical properties such as geometry, U-values, climate data, indoor temperature and energy end-use supply systems (e.g., heating and cooling systems)^13^. Different modeling approaches can be undertaken depending on the available data. Some defined a set of synthetic buildings reflecting building stock averages, others applied a set of “generic” example buildings from the national typologies.

The bottom-up building sectoral modeling studies. The sectoral in this study also provide additional data that are not explicitly presented in the existing publications while layering on additional socioeconomic data from worldwide databases. The method to align the models is composed of three main steps: (1) definition of a common framework, including outcome variables, assumptions, and viable scenarios; (2) collection of modeling results; and (3) comparative visualization and analysis of the results. The study standardizes common parameters from country-specific conditions: start year (2020), target year (2050), time interval (5-year), subsectors (disaggregation to “residential” and other nonresidential services as “commercial”), outcome variables (FEC, CO_2_ emissions, floor area, carbon intensity of electricity production, and shares of electricity in the total demand and heating demand). Country-specific descriptions of the building stock and contextual parameters required as the input in the modeling exercise are as follows: current building structure, building technological systems, fuel mix in the energy supply, CO_2_ emissions of fuels, and policy instruments such as codes, standards and regulations, economic instruments, and information and education programs. Some countries in Europe are addressed by more than one model, and in such cases, we calculate the mean of the results per county. National results are aggregated in accordance with the IPCC division into world regions, similar to standard M49. The results of the bottom-up sectoral models and the mean values for the European clusters are population-weighted.

### Descriptions of participating models

We employed eight state-of-the-art sectoral building stock models, which are briefly described below. Additional details and references can be found in Supplementary Table 2.

Brazilian Land-Use and Energy System model (BLUES) was developed using the MESSAGE platform (Model for Energy Supply System Alternatives and their General Environmental Impacts) by the International Institute for Applied Systems Analysis. It is a mixed-integer linear optimization model, which minimizes the total cost of expanding the energy system to meet an expected demand for energy services. It combines technical, economic, and environmental variables for more than 8,000 technologies with imposed constraints to obtain an optimal solution for the energy sector. The model can have endogenous or exogenous demand responding elastically to the variables of population growth and GDP, which can also respond to price signals. BLUES provides detailed geographical coverage of Brazil and has 1-h time resolution.

CoreBee developed by JPJN and FF is a bottom-up-model that focuses on the cost-optimal renovation of national building stocks. It is based on quasi-steady state assumptions for calculating the energy demand for both heating and cooling. It identifies the cost-optimal renovation options (building envelopes, technical systems, and renewable energy generation) for each national building stock with regards to both primary energy savings and CO_2_ emissions. The model is primarily designed for application to the existing European Union (EU) building stock represented by reference buildings defined by their construction period, typology, envelope insulation levels, and heating and cooling supply systems. It also explores the effects of both the current and future energy system fuel mixes on carbon intensity and the supply of heating and cooling.

China 2050 Demand Resources Energy Analysis Model (DREAM) is a bottom-up energy and emissions analysis model developed by the Lawrence Berkeley National Laboratory in California. The model consists of China’s five energy demand sectors (residential buildings, commercial buildings, industry, transport, and agriculture) and an energy transformation module that includes energy production, transmission, and distribution. The DREAM model is implemented by using the Long-range Energy Alternatives Planning platform. The building sector module captures the building stock turnover to reflect China’s rapid urbanization by 2050. By using China’s major climate zones, the results are segmented to characterize building sector energy use in cold, transition, and warm climate zones. The model allows scenario analysis for different energy saving and emission reduction measures, including energy efficiency retrofits to the existing building stock and implementation of different building codes and standards in new construction. Furthermore, DREAM can model energy and technologies at the end-use level (including fuel type). These energy end uses are connected to DREAM’s transformation module to analyze the primary energy use and CO_2_ emissions of the building sector.

Energy, Carbon and Cost Assessment for Building Stocks (ECCABS) is a bottom-up model used to assess energy conservation measures (ECMs) and CO_2_ mitigation strategies in existing building stocks. The model is based on a one-zone hourly heat balance that calculates the net energy demand for a number of representative buildings and then extrapolates the results to the entire stock via weighting coefficients. The model generates results in terms of net and final energy values, associated CO_2_ emissions, and the costs of implementing different ECMs; these results are then applied according to cost-efficiency over a timeline for a series of scenarios (e.g., the cost of renovations changes in and energy prices).

Ecuador Land-Use and Energy Network Analysis (ELENA) is the first integrated assessment model (IAM) for Ecuador. It was developed by the Escuela Politécnica Nacional with the technical assistance of the Federal University of Rio de Janeiro (COPPE/UFRJ) in Brazil during the DDP-LAC project. ELENA’s^25^ structure is based on the BLUES model^26^ and uses some outputs of the COFFEE model. ELENA assesses from a bottom-up modeling approach the expansion of the energy–land-use system and the evolution of CO_2_ emissions through 2050. It is a partial equilibrium, integrated, optimization model using a perfect foresight approach that considers the whole energy conversion chain, from primary energy to useful energy. Five economic sectors, including buildings, are represented in the model. ELENA also models the land-use system by calculating land-use changes according to the demand for food and deforestation/reforestation scenarios up to 2050. Useful energy and food demands, as well as deforestation/reforestation, are exogenously calculated.

Invert/EE-Lab is a dynamic bottom-up simulation tool that evaluates the effects of different framework conditions (especially different economic and regulatory incentives) on possible evolution of energy demand, energy carrier mix, CO_2_ reductions, and the cost of space heating, cooling, and hot water in buildings. The model describes the building stock and heating, cooling, and hot water systems at highly disaggregated levels, calculates related energy needs and delivered energy, determines reinvestment cycles and new investments of building components and technologies, and simulates the decisions of various agents (i.e., owner types), assuming that an investment decision is due for a specific building segment. The core of the tool is a myopic, multinomial logit approach, which optimizes the objectives of “agents” under conditions of imperfect information and thus represents the decision-maker taking building-related decisions. Although the spatial resolution of the tool is national, climate zones and sub-regions are distinguished for some countries. Furthermore, the energy-demand-related outputs of these tools can be displayed on a 100 m^2^m^2^ raster cell level. The model Invert/EE-Lab has until now been applied to the building stock in all EU-27 (+GBR) countries^1,27–29^.

RE-BUILDS^30^ utilizes dynamic Material Flow Analysis for studying the long-term development of the Norwegian building stock. The driving force in the model is a population’s need for housing and various types of commercial buildings. The demand for floor area in buildings of various types is estimated for each year. Demolition and renovation activities are estimated using probability functions. Floor area is distributed among archetypes based on building type, construction year, and renovation status. Archetype-specific energy intensities, fuel mix, and use of local RESs are used to estimate the aggregated energy demand from the building stock, as well as the use of various energy carriers. Carbon emission intensities per energy carrier are used to estimate the aggregated CO_2_ emissions. The model is used for scenario analyses, wherein various inputs are varied among the scenarios, and in particular, the scenarios that affect the energy intensities assumed for new construction and renovated buildings from various archetypes.

Scout is a bottom-up model of the USA residential and commercial building sectors that estimates the impact of various ECMs on building sector energy demand. This model characterizes ECMs using their relative or absolute energy performance, installed cost, service lifetimes, and year of introduction into the market. Probability distributions can be placed on ECM performance, cost, and lifetime inputs, which then filter through to final energy and carbon impacts. Furthermore, the ECM energy performance can be calculated using whole-building energy simulation via EnergyPlus and can be applied to the Scout prototype building models. This approach compares ECMs on a level playing field using identical assumptions, thus eliminating the need for normalization. It also produces savings estimates disaggregated by end-use, thereby facilitating the evaluation of ECM packages. Scout ECMs are applied to a baseline USA building stock that is defined in the Annual Energy Outlook (AEO)^31,32^ of the Energy Information Administration with the granularity of building type (e.g., office, hospital, and single-family home), building vintage (e.g., new and existing), climate zone (e.g., hot and humid and cold), end-use (e.g., heating and lighting), and fuel type (e.g., electricity and natural gas). Scout also uses the AEO to project growth and stock turnover in each baseline market segment. Various AEO scenarios are used for the renewable penetration of electricity supply for this study.

## Supplementary information

Supplementary Information

Peer Review File

## Data Availability

All data generated or analyzed during this study used in the graphs are included in this published article and its supplementary information files: “Supplementary References 1. Numerical output per figure”. Further data are available from the corresponding author on reasonable request.

## Source data

Source Data
Author Checklist

## References

[1] International Energy Agency (IEA). World Energy Outlook 2021. https://www.iea.org/reports/world-energy-outlook-2021.

[2] Ürge-Vorsatz D, Cabeza LF, Serrano S, Barreneche C, Petrichenko K (2015). Heating and cooling energy trends and drivers in buildings. Renew. Sustain. Energy Rev..

[3] Riahi K (2017). The shared socioeconomic pathways and their energy, land use, and greenhouse gas emissions implications: An overview. Glob. Environ. Chang..

[4] Levesque A (2018). How much energy will buildings consume in 2100? A global perspective within a scenario framework. Energy.

[5] Wang H, Chen W, Shi J (2018). Low carbon transition of global building sector under 2- and 1.5-degree targets. Appl. Energy.

[6] Levesque, A., Pietzcker, R. C., Baumstark, L. & Luderer, G. Deep decarbonization of buildings energy services through demand and supply transformations in a 1.5 °C scenario. *Environ. Res. Lett.* **16**, 054071 (2021).

[7] Grubler A (2018). A low energy demand scenario for meeting the 1.5 °C target and sustainable development goals without negative emission technologies. Nat. Energy.

[8] Levesque A, Pietzcker RC, Luderer G (2019). Halving energy demand from buildings: The impact of low consumption practices. Technol. Forecast. Soc. Change.

[9] Rogelj, J. et al. Chapter 2: Mitigation pathways compatible with 1.5 °C in the context of sustainable development Global Warming of 1.5 °C, an IPCC Special Report on the Impacts of Global Warming of 1.5 °C above Pre-industrial Levels and Related Global Greenhouse Gas Emission Pathways, in the Context of Strengthening the Global Response to the Threat of Climate Change (Geneva: Intergovernmental Panel on Climate Change) (2018).

[10] Luderer G (2018). Residual fossil CO_2_ emissions in 1.5–2 C pathways. Nat. Clim. Change.

[11] Ürge-Vorsatz, D. et al. Advances toward a net-zero global building sector. 10.1146/annurev-environ-012420 (2020).

[12] Mata, É. et al. A map of roadmaps for zero and low energy and carbon buildings worldwide. *Environ. Res.*10.1088/1748-9326/abb69f (2020).

[13] Langevin, J. et al. Developing a common approach for classifying building stock energy models. *Renew. Sustain. Energy Rev.* **133**, 110276 (2020).

[14] Kavgic, M. et al. A review of bottom-up building stock models for energy consumption in the residential sector. *Build. Environ.* **45**, 1683–1697 (2010).

[15] Guo, S., Yan, D., Hu, S. & An, J. Global comparison of building energy use data within the context of climate change. *Energy Build*. **226**, 110362 (2020).

[16] IPCC. Summary for Policymakers. Climate Change 2014, Mitigation of Climate Change. Contribution of Working group III to the fifth assessment report of the Intergovernmental Panel on Climate Change. (Cambridge University Press, 2014).

[17] Mata, É. et al. A systematic map of determinants for buildings’ energy demand and climate impact. *Environ. Res. Lett.* **16**, 55011 (2020).

[18] Reyna JL, Chester MV (2017). Energy efficiency to reduce residential electricity and natural gas use under climate change. Nat. Commun..

[19] Mata É, Wanemark J, Nik VM, Kalagasidis AS (2019). Economic feasibility of building retrofitting mitigation potentials: Climate change uncertainties for Swedish cities. Appl. Energy.

[20] International Energy Agency (IEA). Energy Technology Perspectives 2020. [cited 2021 Aug 20]; Available from: www.iea.org/t&c/.

[21] Viggers H, Keall M, Wickens K, Howden-Chapman P (2017). Increased house size can cancel out the effect of improved insulation on overall heating energy requirements. Energy Policy [Internet]..

[22] Ellsworth-Krebs KatherineL, Hunter CJ (2021). Home comfort and “peak household”: implications for energy demand. housing. Theory Soc. [Internet]..

[23] Cohen MJ (2021). New conceptions of sufficient home size in high-income countries: are we approaching a sustainable consumption transition? housing. Theory Soc. [Internet]..

[24] Millward-Hopkins J, Steinberger JK, Rao ND, Oswald Y (2020). Providing decent living with minimum energy: A global scenario. Global Environ. Change.

[25] Rua Rodriguez Rochedo, P. Development of a global integrated energy model to evaluate the brazilian role in climate change mitigation scenarios. PhD Thesis. COPPE-UFRJ (2016).

[26] Köberle AC, Rochedo PRR, Lucena AFP, Szklo A, Schaeffer R (2020). Brazil’s emission trajectories in a well-below 2 °C world: the role of disruptive technologies versus land-based mitigation in an already low-emission energy system. Clim. Change.

[27] Kranzl L, Hummel M, Müller A, Steinbach J (2013). Renewable heating: Perspectives and the impact of policy instruments. Energy Policy.

[28] Kranzl L (2019). Are scenarios of energy demand in the building stock in line with Paris targets?. Energy Effic..

[29] Hartner, M. et al. H2020 SET-Nav D.5.8: WP5 Summary report—Energy Systems: Demand perspective. (2019).

[30] Sandberg, N. H., Naess, J. S., Brattebø, H., Andresen, I. & Gustavsen, A. Large potentials for energy saving and greenhouse gas emission reductions from large-scale deployment of zero emission building technologies in a national building. *Energy Policy* **152**, 12114 (2021).

[31] Annual Energy Outlook 2020. U.S. Energy Information Administration (2020) https://www.eia.gov/outlooks/aeo/.

[32] U.S. Energy Information Agency. (2019). Annual Energy Outlook 2019 with projections to 2050. Annu. Energy Outlook 2019 Proj. 2050.

[33] Mata É, Kalagasidis AS, Johnsson F (2018). Contributions of building retrofitting in five member states to EU targets for energy savings. Renew. Sustain. Energy Rev..

[34] Müller, A. Energy Demand Assessment for Space Conditioning and Domestic Hot Water: A Case Study for the Austrian Building Stock. 285 http://www.invert.at/Dateien/Dissertation_AndreasM.pdf (2015).

[35] Filippidou, F. & Jiménez Navarro, J. P. Achieving the cost-effective energy transformation of Europe’s buildings Energy renovations via combinations of insulation and heating & cooling technologies: Methods and data. European Commission, Joint Research Centre 10.2760/278207 (2019).

[36] Langevin, J., Harris, C. B. & Janet, R. J. Assessing the Potential to Reduce U.S. Building CO2 Emissions 80% by 2050. Joule 10.1016/j.joule.2019.07.013 (2019).

[37] Rochedo PRR (2018). The threat of political bargaining to climate mitigation in Brazil. Nat. Clim. Change.

[38] Villamar D (2021). Long-term deep decarbonization pathways for Ecuador: Insights from an integrated assessment model. Energy Strateg. Rev..

[39] Wang, R., Lu, S. & Feng, W. A novel improved model for building energy consumption prediction based on model integration. *Appl. Energy* 262 (2020).

