# Supplementary material for: A global comparison of building decarbonization scenarios by 2050 towards 1.5–2 °C targets
Source: Nat Commun. 2022 Jun 2;13:3077. doi: 10.1038/s41467-022-29890-5 (PMC9163154; doi:10.1038/s41467-022-29890-5)
Supplement: Supplementary file 1 — Supplementary Information [file 41467_2022_29890_MOESM1_ESM.docx]

A global comparison of building decarbonization scenarios by 2050 towards 1.5 -2 °C targets

# Supplementary Table 1. Comparative description of the modeled national building sectors

Supplementary Table 1. Comparative description of the national building sectors modeled as per the reference scenario (RS) in 2020.

| **Region** | **Country** | **Population** | **Land area** | **Population density** | **U-value (baseline)** | **GDP** | **GDP** | **HFA** | **HDD** | **CDD** | **Climate severity (HDD + CDD)** |
| --- | --- | --- | --- | --- | --- | --- | --- | --- | --- | --- | --- |
|  |  | **(million)** | **(km^2^)** | **(hab./km^2^)** | **(W/m²K)** | **(Billion US$2020*/yr)** | **(US$/capita)** | **(Million m^2^)** | **(n)** | **(n)** |  |
| NW Europe | DEU | 82 | 357,386 | 229 | CoreBee: U-value per envelope component (wall, roof, floor, window, and door). For the renovated German building stock: 0.2, 0.2, 0.2, 1, and 1, respectively. For the renovated Greek building stock: 3.05, 2.2, 3.1, 4.1, and 1.5, respectively | 2,690 | 32,846 | 3,199 | 3,032 | 23 | 3,055 |
|  | FRA | 67 | 643,801 | 103 | In Invert/ EE-lab model; Wall: 0, 1–0, and 3; roof: 0, 1–0, and 2; floor: 0, 1–0, and 3; window: 0, 9–2, and 3 | 2,540 | 38,130 | 2,486 | 2,354 | 47 | 2,401 |
|  | GBR | 66 | 242,495 | 273 |  | 2,760 | 41,692 | 3,330 | 3,020 | 0 | 3,020 |
|  | DNK | 6 | 42,933 | 138 |  | 370 | 64 | 390 | 3,245 | 2 | 3,247 |
|  | SWE | 10 | 450,295 | 23 |  | 452 | 44,422 | 700 | 5,225 | 1 | 5,226 |
|  | NOR | 5 | 385,203 | 14 | RE-BUILDS: Only indirectly considered: Study uses the average energy intensity for different archetypes taken from other projects | 318 | 58,609 | 370 | 3603 | 0 | 3603 |
|  | EST | 1 | 45,227 | 29 | In Invert/ EE-lab model; wall: 0, 1–0, and 3; roof: 0, 1–0, and 2; floor: 0, 1–0, and 3; window: 0, 9–2, and 3 | 33 | 25 | 80 | 4,243 | 10 | 4,253 |
|  | FIN | 6 | 338,455 | 17 |  | 277 | 49 | 340 | 5,468 | 3 | 5,471 |
|  | IRL | 5 | 84,421 | 60 |  | 217 | 43 | 230 | 2,816 | 0 | 2,816 |
|  | LTU | 3 | 65,300 | 49 |  | 107 | 34 | 120 | 3,868 | 17 | 3,885 |
|  | AUT | 9 | 83,879 | 104 |  | 461 | 53 | 600 | 3,456 | 29 | 3,485 |
|  | BEL | 11 | 30,689 | 367 |  | 550 | 49 | 560 | 2,678 | 18 | 2,696 |
|  | LUX | 1 | 2,586 | 228 |  | 67 | 113 | 40 | 2,881 | 28 | 2,909 |
|  | LVA | 2 | 64,589 | 33 |  | 63 | 30 | 70 | 4,062 | 12 | 4,074 |
|  | NLD | 17 | 41,865 | 412 |  | 1,004 | 58 | 800 | 2,704 | 12 | 2,716 |
| SE Europe | ESP | 49 | 505,990 | 96 | In the Invert/EE-lab; wall: 0, 1–0, and 6; roof: 0, 1–0, and 4; floor: 0, 2–0, and 6; window: 1–2 (upper value representing the 75% quantile of all cases) | 2,016 | 41 | 1,270 | 1,735 | 246 | 1,981 |
|  | BGR | 11 | 110,993 | 103 |  | 70 | 6 | 190 | 2,485 | 165 | 2,650 |
|  | CYP | 1 | 9,251 | 135 |  | 36 | 29 | 40 | 665 | 703 | 1,368 |
|  | GRC | 11 | 131,957 | 87 | CoreBee: U-value per envelope component (wall, roof, floor, window, and door). For the renovated German building stock: 0.2, 0.2, 0.2, 1, and 1. For the renovated Greek building stock: 3.05, 2.2, 3.1, 4.1, and 1.5 | 393 | 34 | 580 | 1,534 | 331 | 1,865 |
|  | ITA | 62 | 301,340 | 205 | In the Invert/EE-lab; wall: 0, 1–0, and 6; roof: 0, 1–0, and 4; floor: 0, 2–0, and 6; window: 1–2 (upper value representing the 75% quantile of all cases) | 2,443 | 40 | 3,360 | 1,861 | 232 | 2,093 |
|  | MLT | 0 | 316 | 1,361 |  | 1989 | 33 | 10 | 470 | 627 | 1,097 |
|  | PRT | 11 | 92,226 | 118 |  | 372 | 34 | 420 | 1,204 | 206 | 1,410 |
|  | SVN | 2 | 20,271 | 103 |  | 83 | 40 | 100 | 2,765 | 57 | 2,822 |
|  | CZE | 11 | 78,866 | 139 |  | 262 | 24 | 410 | 3,288 | 30 | 3,318 |
|  | HUN | 10 | 93,030 | 105 |  | 350 | 36 | 450 | 2,660 | 113 | 2,773 |
|  | POL | 38 | 312,696 | 123 |  | 1,353 | 35 | 1,520 | 3,365 | 27 | 3,392 |
|  | SVK | 6 | 49,035 | 114 |  | 209 | 37 | 200 | 3,161 | 52 | 3,213 |
| North America | USA | 336 | 9,834,000 | 34 | Overall U-value is not available; the envelope mix is determined using a bottom-up methodology that competes multiple envelope performance tiers | 15,068 | 44,879 | 28,100 | 1,987 | 1,547 | 3,533 |
| South America and Caribbean | ECU | 16 | 283,560 | 61 | Not considered. It is a bottom-up model to simulate the whole national energy conversion chain. The demand for energy services is driven by top-down sociotechnical considerations | 136 | 8,339 | 343 | 0 | 224 | 224 |
|  | BRA | 211 | 8,516,000 | 25 | Not considered. The model uses a bottom-up socioeconomical methodology | 2,582 | 12,250 | 1,522 | 180 | 1,846 | 2,026 |
| Eastern Asia | CHN | 1,414 | 9,706,961 | 146 | Not explicitly modeled. For new construction, please refer to China’s net-zero energy building standards and U-values requirement for different climate zones- GB/T51350-2019 | 10,613 | 7,506 | 46,610 | 4,267 | 73 | 4,340 |
| *Original data 2006 | | | | | | | | | | | |

# Supplementary Table 2. Modeling and scenarios

## 2.1. Key assumptions per model and scenario: technology and demand developments, policy instruments, and inputs

Supplementary Table 2.1. Key assumptions used per model and scenario. Technology and demand development: DR stands for demand reduction; EE, energy efficiency; RO, renewable onsite; RG, renewable grid; and EL, electrification.

| Model | | Scenario | Technology and demand development* | Policy instruments | Energy price | Demographic and floor area development | Demand drivers | Primary model inputs |
| --- | --- | --- | --- | --- | --- | --- | --- | --- |
| Scout/AEO (USA) |  | RS  (AEO2019-Ref) | D/EE: reference case building technology development  RG: reference electricity supply projections  EL: no electrification incentives | None | Reference energy supply prices (national macroeconomic model) | Baseline USA population growth projections | Building equipment capital cost and energy cost, building stock growth, floor area per residence | Techno-economics |
|  |  | DS  (AEO2019-SDS, AEO2019-HR) | D/EE: mix of the reference case, best commercially available, and targeted high-efficiency: heating, cooling, lighting, envelope, water heating, refrigeration, and appliances  RG: Higher renewable power supply achieved via $25/t CO_2_ carbon tax.  EL: electrification with 20% capital cost credit | 20% capital cost credit for electrification  $25/t CO_2_ carbon tax | Reference energy supply prices (national macroeconomic model) | Baseline US population growth projections | Building equipment capital cost and energy cost | Techno-economics |
| DREAM (CHN) | RS  (Reference) | | RG: Reference electricity supply projections EE/RO: Currently available building technologies EL: no incentives | Continuing existing policy adoption at its current pace | Maintain the same as today | Maintain the same as today | Not taken into account | Baseline population growth rate and 80% urbanization rate in 2050. Per capita living space and working space increase over years. |
|  | DS (Electrification) | | RG: High renewable penetration in the power sector  DR/EE: Aggressive building EE policies for new and existing buildings EE increase  EL: Enhance fuel switch to EL | 1) Promote net-zero energy buildings; 2) retrofit existing buildings; 3) use high-efficiency appliances in buildings; and 4) renewable energy penetration in buildings | Maintain the same as today | Baseline population growth rate and 80% urbanization rate in 2050. Per capita living space and working space also increase over years | Not taken into account | Same as above but different parameter values for the energy load demand, EE, technology shares, and stock for retrofit and low-energy new construction. |
|  | DS  (High Electrification) | RG: 1) High renewable penetration in the power sector  DR/EE: 2) Aggressive building energy efficiency BEE policies for new and existing buildings; 3) Technology efficiency improve and enhance fuel switch for electrification  EL: 4) Improve electrification rate for heating, water heating, and cooking. Overall social scale electrification rate reaches 70% in 2050 | | 1) Promote net-zero energy buildings; 2) retrofit existing buildings; 3) use high-efficiency appliances in buildings; 4) renewable energy penetration in buildings; and 5) electrification of building technologies for heating, water heating, and cooking | Maintain the same as today | Baseline population growth rate and 80% urbanization rate in 2050. Per capita living space and working space also increase over years |  | Same as the high-efficiency scenario with changes in the technology share to enhance end-use technology electrification |
| ECCABS (SWE, FRA, DEU, ESP, GBR)  (Only SWE) |  | RS (BAU-TE) | Same choices for RS and DS, only the uptake changes.  DR: thermostats to 20C^*^; EE: Improved building envelope, ventilations with heat recovery, improved efficiency of lighting and appliances; RO: photovoltaic (PV) panels^*^, solar hot water, biomass boilers; RG: n.c.; EL: n.c.  U-values in the range 0.5–1.9 for R and 0.8–1.9 for C, depending on the country | Existing measures, assumed to favor cost effective measures as today. | Maintain the same as today | Not taken into account | Climate, characteristics of buildings and technical systems, occupant needs | Techno-economic |
|  |  | DS (BAU-T) |  | Assumed to favor full deployment of technical potentials | Maintain the same as today | Not taken into account | Climate, characteristics of buildings and technical systems, occupant needs | Technical |
| RE-BUILDS (NOR) |  | RS (Baseline) | DR: New construction after 2025 assumed as passive houses. Energy intensity of renovated buildings follows the recent trends for various stock segments.  RO slowly introduced | Only applied indirectly, as the extent to which the technology improvements are introduced varies between scenarios. This can result from policy instruments (e.g., regulations related to the energy demand of new construction), but the instruments are not taken directly into account. | Not taken into account | Population statistics and medium scenario for future population projections from Statistics Norway | Number of persons per dwelling. Floor area per dwelling. Floor area per person in nonresidential buildings. | Key model inputs: Population statistics and projections, lifestyle parameters, renovation and demolition probability functions, archetype-specific energy intensities, energy mix, and assumptions on the use of local renewable energy sources (RESs). RS assumes a continuation of trends in the energy performance levels of new and renovated buildings as well as in the use of local RESs. Five alternative emission intensities for electricity are applied and compared. |
|  |  | DS (Ambitious Zero-emission Building scenario) | DR: The concept of zero-emission building is rapidly introduced in the new construction. Advanced renovation with best available solutions for energy savings is assumed for all stock segments.  RO: Rapidly introduced | Only applied indirectly, as the extent to which the technology improvements are introduced varies between scenarios. This can result from policy instruments (e.g., regulations related to the energy demand of new construction or subsidy schemes for renovation), but the instruments are not taken directly into account.  U-values only indirectly considered: The study uses average energy intensity for different archetypes taken from other projects | Not taken into account | Population statistics and medium scenario for future population projections from Statistics Norway | The demand for floor area of various types is assumed equal in both scenarios. | Key model inputs as in RS. DS assumes a large-scale and rapid introduction of zero-emission buildings in new construction, with the ambition level increasing over time and large-scale use of advanced renovation with high energy efficiency by using the already available zero-emission building technology. The frequency of construction and renovation is equal in both scenarios. Construction is according to estimated demand, and renovation is according to the simulated “natural” need for renovation resulting from the aging of the stock and the need for maintenance. Five alternative emission intensities for electricity are applied and compared as in the RS. |
| CoreBee (DEU, GRE) |  | RS (1%_RR) | DR: Prosumer strategies for micro combined heat and power (μCHP)  EE: Upgrades for the building envelope and ventilation and the heating and cooling technical systems (including SH, SC, and DHW)  RO: Small-scale (solar thermal and PV systems)  RG: n.c.  EL: n.c. | In line with the European Commission cost-optimal and long-term renovation strategy for buildings  Guidelines laid down in the EPBD (2018/844) | Not taken into account | Not taken into account | (input data)  Climate  Building characteristics and energy needs  Technical building systems (efficiencies and costs)  Energy costs  National fuel mixes  Renovation rates (1%/yr)  Capital expenditure (CAPEX) and operations expenditure (OPEX) of renovation solutions | Techno-economics |
|  |  | DS (2%_RR, 3%_RR) | DR: Prosumer strategies for μCHP  EE: Upgrades of building envelope and ventilation and heating and cooling technical system (including SH, SC, and DHW); U-value per envelope component (wall, roof, floor, window, and door); for the renovated German building stock: 0.2, 0.2, 0.2, 1, and 1, respectively; for the renovated Greek building stock: 3.05, 2.2, 3.1, 4.1, and 1.5, respectively.  RO: Small-scale (solar thermal and PV systems)  RG: n.c.  EL: n.c. | In line with the European Commission cost-optimal and long-term renovation strategy for building  Guidelines laid down in the EPBD (2018/844) | Not taken into account | Not taken into account | (input data)  Climate  Building characteristics and energy needs  Technical building systems (efficiencies and costs)  Energy costs  National fuel mixes  Renovation rates (3%/yr)  CAPEX and OPEX of renovation solutions | Techno-economics |
| Invert/EE-Lab (EU-28) |  | RS (Reference) | Current policy scenario for each country | • Current policy scenario for each country | • Wholesale fossil—IEA scenario 2016, electricity—Enertile© model, retail prices by Fraunhofer ISI (including CO_2_ price) | • Population growth projections per country from Primes EU Reference Scenario 2016 | • Specific heated floor area • Development of building stock • Thermal building quality • Investment decisions • User behavior | Main existing policies: building codes, energy taxation, and support schemes |
|  |  | DS–(Diversification) | RO/EL: Decentralized technology mix (heat pumps, biomass boilers, and solar thermal systems) and smart heating  RO/EL/RG: Phasing out fossil fuel (natural gas from 2030) | • Prohibition of new fossil fuel heating systems from 2030 • High subsidies for heat pumps • Support for smart thermostats | • Wholesale fossil—IEA scenario 2016, electricity—Enertile© model, retail prices by Fraunhofer ISI (including CO_2_ price) | • Population growth projections per country from Primes EU Reference Scenario 2016 | • Specific heated floor area • Development of the building stock • Thermal building quality • Investment decisions • User behavior | Stricter regulatory policy instruments, in particular regarding the obligation for installing RES-H systems in case of heating system replacement after 2030 |
|  |  | DS (Directed vision) | EE: Concerted thermal renovation  RG: Coherent district heating uptake planning  RO/EL: Decentralized renewable heating in low-heat-density areas  RO/EL/RG: Phasing out fossil fuel (natural gas from 2030) | • Deep renovation subsidies and obligations • Zoning, obligations, cost reduction and investment subsidies for district heating | • Wholesale fossil–IEA scenario 2016, electricity–Enertile© model, retail prices by Fraunhofer ISI (including CO_2_ price) | • Population growth projections per country from Primes EU Reference Scenario 2016 | • Specific heated floor area • Development of the building stock • Thermal building quality • Investment decisions • User behavior | Focus on energy efficiency policies in terms of renovation obligations and financial support. Promotion of district heating through more stringent spatial heat planning policies, including the assumption that these policies are effective in promoting district heating. |
|  |  | DS  (2DS–Localization) | RG/EE: Focus on national resources  RO: Higher diffusion of solar thermal and PV systems  RO: Strong biomass use  RO/EL/RG: Phasing out fossil fuels | • Enhanced PV and solar thermal subsidies • Moderate support of national resources usage | • Wholesale fossil –IEA scenario 2016, electricity –Enertile© model, retail prices by Fraunhofer ISI (including CO_2_ price) | • Population growth projections per country from Primes EU Reference Scenario 2016 | • Specific heated floor area • Development of building stock • Thermal building quality • Investment decisions • User behavior | Focus on local resources, in particular solar energy, i.e., stronger financial support for PV and solar thermal systems. |
|  |  | DS  (National champions) | RO/EL/RG: No concerted phasing out of fossil fuels (green gas as an option); Different strategies depending on the country | • Country-dependent strategies based on current status, and partial green gas promotion | • Wholesale fossil–IEA scenario 2016, electricity–Enertile© model, retail prices by Fraunhofer ISI (including CO_2_ price) | • Population growth projections per country from Primes EU Reference Scenario 2016 | • Specific heated floor area • Development of building stock • Thermal building quality • Investment decisions • User behavior | Main focus of energy carriers remains in place. That is, in countries with currently a high gas share, green gas plays a stronger role, whereas in countries with a high share of district heating, decarbonized district heating etc. play an important role. Hence, country-specific policies were implemented in the model. |
| BLUES_v2.0 (BRA) |  | RS | For all scenarios: U-values are not considered.  The model uses a bottom-up socioeconomic methodology | RS does not have policy instruments, and for all other policies, emissions tax is used. | Not taken into account | Demographic development accordingly to SSPs narratives. The model did not use floor area as a parameter | Population, GDP, energy intensity, and technological choices/cost | Techno-economics |
|  |  | Adb | DR: n.c.  EE: n.c.  RO: n.c.  RG: n.c.  EL: n.c. |  |  |  |  |  |
|  |  | ssp1_bau | DR: Demand reduction of 0.5%/yr  EE: Efficiency gains in lighting technologies and electrical appliances.  RO: Financial incentive for small-scale implementation (solar thermal and PV systems).  RG: Greater share of renewables in the electricity grid.  EL: n.c. for the residential sector (only for vehicles) |  |  |  |  |  |
|  |  | ssp3_bau | DR: n.c.  EE: Efficiency gains in lighting technologies and electrical appliances  RO: n.c.  RG: Greater share of renewables in the electricity grid.  EL: n.c. |  |  |  |  |  |
|  |  | ssp4_bau | DR: n.c.  EE: Efficiency gains in lighting technologies and electrical appliances.  RO: Financial incentive for small-scale implementation (solar thermal and PV systems).  RG: Greater share of renewables in the electricity grid.  EL: n.c. for the residential sector (only for vehicles) |  |  |  |  |  |
|  |  | ssp5_bau | DR: n.c.  EE: Efficiency gains in lighting technologies and electrical appliances  RO: n.c.  RG: n.c.  EL: n.c. |  |  |  |  |  |
|  |  | DS | No technological changes from RS scenarios. Only carbon budgets aligned with 2DS | Emissions tax | Not taken into account | Demographic development according to SSPs narratives. The model did not use floor area as a parameter | Population, GDP, energy intensity, and technological choices/costs | Techno-economics and four scenarios according to SSP narratives, SSP1, 3, 4, and 5, with carbon budget limits for all sectors together of SSP1 = 29.2 Gt CO_2_, SSP3 = 25.5 Gt CO_2_, SSP4 = 21.9 Gt CO_2_, SSP5 = 31.3 Gt CO_2_ |
| ELENA (ECU) |  | RS (RS) | Same choices for the RS and DS, only the uptake changes.  EE: lightning, cooking, water heating, air cooling, and electric appliances38 | Existing policies, e.g., the National Plan for Energy Efficiency (PLANEE) and the Master Electrification Plan (PME). | The model constructs the cost of each energy source along the transformation chain. It is not a pricing model | GDP, population growth, household size, and floor space per capita scenarios up to 2050 | Number of households and people for residential buildings.  Commercial GDP for commercial buildings. | RS scenario considers a least-cost expansion subject to ongoing energy policies. In the power sector, it includes thermoelectric plants powered by fossil fuels and hydropower plants, and fossil fuels are the main fuel used in the transport sector.  DDP is restricted by a cumulative carbon budget of 1.46 GtCO_2_ for the period 2010–2050, in compliance with the Paris Agreement. |
|  |  | DS (Deep Decarbonization Pathways, DDP) | EL: to cook with induction stoves. Water heating with electric showers and boilers.  RO: for water heating demand. PV distributed generation.  EE: U-values in DS are not considered. ELENA is a bottom-up model to simulate the whole national energy conversion chain, energy services demand is driven by top-down sociotechnical considerations | PLANEE and PME policies. Efficient cooking program | Same as RS | Same as RS. And developed countries’ energy consumption were taken as goals for 2050 in | Same as RS |  |
| *Hydrogen has not been considered as multiple studies show that it is not a viable option when it comes to heating buildings. The amount of green electricity needed to produce green hydrogen for this purpose is 500% to 600% greater than the amount needed to power an equivalent number of heat pumps^40, 41^. Hydrogen is acknowledged as a relevant fuel in sectors such as transport or industry^41^.  **ADS assumptions may be conservative about the prospect of commercial technology improvement and fuel switching over the long run, particularly in the context of the current US policy discussions about promoting technological improvements to meet new climate goals. | | | | | | | | |

## 2.2. Key drivers of the models

Supplementary Table 2.2. Key drivers of the models: new construction versus retrofit rates, and what is needed to attain complete decarbonization of the building sector.

|  | **ECCABS** | **CoreBee** | **RE-BUILDS** | **Invert/ EE-lab** | **Scout** | **BLUES v2.0** | **ELENA** | **DREAM** |
| --- | --- | --- | --- | --- | --- | --- | --- | --- |
| **Drivers for model results** | The model results are driven by (i) the amount and type of the energy saving measures considered and their costs, (ii) the assumed interest rates and lifetime of the components, and (iii) the energy prices and carbon intensity of the different energy carriers. Great cost variations are found for all buildings. Measures to reduce the energy requirements are typically more cost efficient and climate resilient, but comprehensive deep renovation packages also including efficient appliances and RES are more effective in terms of both cost and mitigation. | CoreBee model explores how different levels of targeted renovations rates will reduce both energy consumptions and associated CO_2_ emissions of the EU building stock toward 2050. It looks into EU energy policy targets (3%) and applies it over time. Therefore, it focuses on existing building under the assumption that renovation is the key pillar for the energy transformation of buildings in Europe. CoreBee allows identifying the segment of the national building stock defined by age and typology that offers higher potentials. | RE-BUILDS model results estimate the technical potential for energy and carbon emission savings from large-scale introduction of zero-emission buildings (ZEB) and ZEB technologies. The RS results are driven by population growth, lifestyle parameters, building stock dynamics, and assumption on the continuation of trends. In the DSs, the energy reductions are additionally driven by assumptions that all new construction are ZEB and that all renovated buildings will apply the best available energy savings technologies. This is theoretically possible, though unrealistic. | The main drivers of the Invert/EE-Lab model are the growth of building stock, costs, and policies affecting effective costs of different technologies, and renovation measures, regulatory policies (such as building codes or RES-H obligations), resource potentials, and assumptions on investment and operational behavior. Invert/EE-Lab does not include other sectors, so there is no way of directly comparing decarbonization costs with those of the other sectors (and this was not the intention of this study). The prosumer choice on the mix of different decarbonization measures (such as building different renovation depths or choice of heating systems) is reflected by a multinomial logit model that determines the market share of different options. | Population growth causes general increases in the AEO/Scout models, but new, higher-efficiency technologies reduce the per capita consumption across all scenarios. In the DS, a credit for fuel switching helps remove onsite fossil fuel burning, which also helps the electricity sector reduce its carbon intensity, through the adoption of increased amounts of renewable generation sources. | Owing to the necessity of decarbonizing the economy as a whole to achieve climate agreements, the model tends to migrate to the development of low-carbon technologies (buildings and others) or enable the storage of atmospheric carbon (energy sector). In the buildings sector, the Brazilian model tends to associate efficiency gains, through changes from traditional fuels to electricity or more efficient fuels, with the generation of distributed electricity (DG) in homes. This becomes important both for the internal consumption of the building sector and for the distribution of this energy on the grid. | ELENA results are basically driven by increase in population, sectoral GDPs, and energy intensities, which affect the demands for energy services, in each sector. As ELENA optimizes the expansion of the energy–land system based on the total minimum cost, the results also depend on the premises of investment and the operation and maintenance (O&M) cost of each available technology. In the DS, the results are additionally driven by a national carbon budget aligned with a 1.5C scenario to achieve the Paris Agreement goals by 2050, as estimated by a global model. Thus, decarbonization decisions in the buildings sector in fact competing with all the other decarbonization options in the other sectors. Our results for buildings come from an integrated national long-term modeling. | Several factors drive the energy consumption and CO_2_ emission in China's building sector. Urbanization and new construction floor space added to China’s building stock is a key factor for the increase in building energy demand. The continuous increase of the service level and thermal comfort of buildings also drives the increase in energy use intensity in buildings. To reduce the building energy demand and CO_2_ emission, a few mitigation factors are considered in our model: (1) Efficient new construction and retrofitted existing buildings will reduce buildings’ loads. (2) The application of highly efficient technologies in buildings will further reduce building energy demand. (3) Building technology fuel switching and electrification, together with high penetration of renewable energy technologies will mitigate the increase in building energy demand. |
| **Insight beyond findings that have already been widely discussed in terms of decarbonizing buildings (i.e., energy efficiency and decarbonization of energy are critical)*** | Renovation of existing buildings, for which an improved building envelope is the most resilient measure against climate change, is not happening fast enough because of constraints related to costs and workmanship capacity. There are substantial additional potentials for mitigation, as decarbonization by GD is not considered in the modeling, and decarbonization that by OD only includes PV systems for Sweden. Although flexibility is required to decarbonize electricity, a series of challenges remain. | Within the European context, it focuses on the renovation of buildings based on their typology and age, and it applies different renovation rates. CoreBee also takes into account the evolution of the energy supply side toward 2050. | Existing buildings will still dominate the Norwegian building stock and its energy use in 2050. There is a larger absolute energy saving potential in renovated buildings than in new construction. Energy use in the Norwegian building stock is dominated by electricity throughout the period in all scenarios. Five alternative emission intensities for electricity are applied. The chosen intensity strongly affects the overall results of the carbon emission saving potential. | When ensuring that direct electric resistance heating is phased out and that heat pumps are used in well-insulated buildings with low inlet temperature levels, a strong increase in the usage of heat pumps is feasible without an increase in the electricity consumption. District heating can play a strong role but requires coherent infrastructure planning and policies. Because of efficiency gains, with the same amount of biomass that is currently used, a higher share of floor area can be heated. However, the question of biomass allocation between sectors remains a policy question, which is not the focus of this study. | A large portion of the US, particularly, in the coldest climates, still uses fossil fuel-based onsite heating. Electrifying these regions with energy efficient options will be a key strategy, as will be improving the thermal shells of the existing building stock. Furthermore, the population in the US continues to grow, so new building codes along with building retrofits will be important strategies for decarbonization. | The building sector in Brazil presents a great opportunity for decarbonization by reducing the use of fuels with low-thermal efficiency for cooking, moving from wood to natural gas. However, there is no migration to electric stoves because of the high cost of electricity in the country. Another highlight is the low importance of converting hot water production from natural gas to electricity, in contrast to the situation in European countries, mainly because electricity-based water heating technology is already used on a large-scale in Brazil. | Ecuador has a good experience with national programs of energy efficiency, for example, replacing LPG stoves by electric induction stoves; replacing old refrigerators by new, efficient ones; and replacing incandescent lamps by efficient ones (e.g., LED). The main opportunity in the buildings sector is related to the replacement of LPG by electric induction stoves. More than 90% of the electricity is produced from RESs. Nevertheless, the cost of induction stoves, special pots, additional connections, etc., is still high. So, far, around 700,000 induction stoves are being used in Ecuador, most of them using subsidized electricity. The challenge is to pursue this goal while removing subsidies. Ecuador also has a high potential to replace LPG by electricity in water -heating applications. In 2010, 60% of the energy used for water heating in the residential sector was from LPG. | Electrification of heating, water heating, and cooking is essential to decarbonize the building sector CO_2_ emission. Electrification should not only consider technology innovation and improvement but also use policies measures to solve issues such as electricity rate reform, expansion of power distribution systems, familiarize building users with new technologies, and leverage electricity market and demand response |

# Supplementary Table 3. Carbon emission factors and share of electricity per country

## 3.1. Carbon emission factor in the RS and DS

Supplementary Table 3.1. Carbon intensity of electricity production and the total energy consumption of the building sector per country in the reference scenarios (RSs). The sources for assumptions regarding electricity are listed in the table, whereas the totals were calculated from the result.

| **Country** | **CO_2_ intensity of electricity production (tCO_2_/MWh)** | | | **Source** | **CO_2_ intensity of total energy consumption (tCO_2_/MWh)** | | |
| --- | --- | --- | --- | --- | --- | --- | --- |
|  | **2020** | **2030** | **2050** |  | **2020** | **2030** | **2050** |
| AUT | 0.15 | 0.10 | 0.06 | ^42^ | 0.15 | 0.14 | 0.09 |
| BEL | 0.14 | 0.28 | 0.10 | ^42^ | 0.20 | 0.19 | 0.16 |
| BGR | 0.48 | 0.27 | 0.06 | ^42^ | 0.23 | 0.17 | 0.13 |
| CYP | 0.61 | 0.47 | 0.18 | ^42^ | 0.30 | 0.26 | 0.16 |
| CZE | 0.51 | 0.38 | 0.05 | ^42^ | 0.21 | 0.16 | 0.14 |
| DNK | 0.39 | 0.26 | 0.06 | ^42^ | 0.19 | 0.16 | 0.09 |
| EST | 0.96 | 0.29 | 0.09 | ^42^ | 0.33 | 0.19 | 0.10 |
| FIN | 0.18 | 0.14 | 0.03 | ^42^ | 0.17 | 0.15 | 0.07 |
| FRA | 0.02  0.05 | 0.03  0.05 | 0.02  0.05 | ^17, 42^ | 0.14  0.14 | 0.14  0.13 | 0.14  0.11 |
| DEU | 0.38  0.58 | 0.30  0.58 | 0.05  0.58 | ^17, 42^ | 0.20  0.31  0.21 | 0.20  0.31  0.19 | 0.20  0.31  0.16 |
| GRC | 0.43 | 0.26 | 0.06 | ^42^ | 0.20  0.27 | 0.20  0.22 | 0.20  0.18 |
| HUN | 0.31 | 0.07 | 0.01 | ^42^ | 0.16 | 0.13 | 0.11 |
| IRL | 0.35 | 0.20 | 0.05 | ^42^ | 0.26 | 0.22 | 0.16 |
| ITA | 0.35 | 0.32 | 0.07 | ^42^ | 0.16 | 0.16 | 0.14 |
| LVA | 0.34 | 0.23 | 0.14 | ^42^ | 0.10 | 0.09 | 0.05 |
| LTU | 0.30 | 0.28 | 0.07 | ^42^ | 0.13 | 0.12 | 0.09 |
| LUX | 0.06 | 0.17 | 0.09 | ^42^ | 0.20 | 0.18 | 0.15 |
| MLT | 0.35 | 0.36 | 0.24 | ^42^ | 0.41 | 0.32 | 0.24 |
| NLD | 0.35 | 0.30 | 0.12 | ^42^ | 0.19 | 0.18 | 0.17 |
| NOR ^a^  NOR ^b^  NOR ^c^  NOR ^d^  NOR ^e^ | 0.02  0.03  0.14  0.31  0.53 | 0.02  0.02  0.14  0.22  0.53 | 0.02  0.01  0.14  0.05  0.53 | ^43^  ^44^  ^43^  ^44^  ^45^ | 0.02  0.03  0.12  0.27  0.46 | 0.02  0.02  0.12  0.19  0.46 | 0.02  0.01  0.12  0.04  0.46 |
| POL | 0.76 | 0.44 | 0.14 | ^42^ | 0.27 | 0.22 | 0.17 |
| PRT | 0.29 | 0.16 | 0.02 | ^42^ | 0.15 | 0.10 | 0.08 |
| ROM | 0.41 | 0.27 | 0.06 | ^42^ | 0.12 | 0.11 | 0.09 |
| SVK | 0.11 | 0.10 | 0.08 | ^42^ | 0.21 | 0.19 | 0.15 |
| SVN | 0.27 | 0.08 | 0.05 | ^42^ | 0.15 | 0.11 | 0.09 |
| ESP | 0.21  0.64 | 0.17  0.64 | 0.08  0.64 | ^17,42^ | 0.42  0.15 | 0.42  0.12 | 0.40  0.11 |
| SWE | 0.03  0. 02 | 0.04  0. 02 | 0.01  0. 02 | ^17,42^ | 0.05  0.15 | 0.05  0.15 | 0.03  0.10 |
| GBR | 0.23  0.48 | 0.18  0.48 | 0.02  0.48 | ^17,42^ | 0.30  0.22 | 0.30  0.20 | 0.30  0.17 |
| USA | 0.43 | 0.39 | 0.34 | ^36^ | 0.30 | 0.28 | 0.26 |
| BRA |  |  |  |  | 0.07 | 0.05 | 0.04 |
| ECU | 0.20 | 0.23 | 0.26 | ^46^ | 0.14 | 0.15 | 0.15 |
| CHN | 0.54 | 0.48 | 0.09 | ^47^ | 0.48 | 0.47 | 0.38 |

Table SM3.2. Carbon intensity of the total energy consumption of the building sector per country in the decarbonization scenarios (DSs) for the year 2050. The sources for assumptions regarding electricity are given in the table, whereas the totals were calculated from the result.

| **Country** | **Source** | **CO_2_ intensity of total energy consumption (tCO_2_/MWh)** | | |
| --- | --- | --- | --- | --- |
|  |  | **Total** | **Commercial** | **Residential** |
| AUT | ^42^ | 0.04 | 0.03 | 0.05 |
| BEL | ^42^ | 0.05 | 0.06 | 0.05 |
| BGR | ^42^ | 0.01 | 0.01 | 0.01 |
| CYP | ^42^ | 0.02 | 0.02 | 0.02 |
| CZE | ^42^ | 0.03 | 0.03 | 0.03 |
| DNK | ^42^ | 0.003 | 0.008 | 0.001 |
| EST | ^42^ | 0.01 | 0.01 | 0.01 |
| FIN | ^42^ | 0.01 | 0.01 | 0.01 |
| FRA | ^17, 42^ | 0.04  0.02 | 0.05  0.02 | 0.04  0.02 |
| DEU | ^17, 42^ | -  -  0.02 | -  -  0.02 | 0.15  0.47  0.02 |
| GRC | ^42^ | -  0.02 | -  0.01 | 0.12  0.03 |
| HRV | ^42^ | 0.01 | 0.01 | 0.01 |
| HUN | ^42^ | 0.03 | 0.03 | 0.03 |
| IRL | ^42^ | 0.03 | 0.03 | 0.03 |
| ITA | ^42^ | 0.01 | 0.02 | 0.01 |
| LVA | ^42^ | 0.01 | 0.01 | 0.01 |
| LTU | ^42^ | 0.01 | 0.01 | 0.01 |
| LUX | ^42^ | 0.05 | 0.07 | 0.03 |
| MLT | ^42^ | 0.03 | 0.05 | 0.03 |
| NLD | ^42^ | 0.05 | 0.05 | 0.05 |
| NOR ^a^  NOR ^b^  NOR ^c^  NOR ^d^  NOR ^e^ | ^43^  ^44^  ^43^  ^44^  ^45^ | 0.02  0.01  0.12  0.04  0.47 | 0.02  0.01  0.12  0.04  0.45 | 0.02  0.02  0.16  0.06  0.63 |
| POL | ^42^ | 0.02 | 0.01 | 0.02 |
| PRT | ^42^ | 0.004 | 0.004 | 0.004 |
| ROM | ^42^ | 0.02 | 0.02 | 0.02 |
| SVK | ^42^ | 0.03 | 0.03 | 0.03 |
| SVN | ^42^ | 0.02 | 0.02 | 0.02 |
| ESP | ^17,42^ | 0.37  0.02 | 0.59  0.01 | 0.25  0.02 |
| SWE | ^17,42^ | 0.04  0.002 | 0.03  0.002 | 0.05  0.002 |
| GBR | ^17,42^ | 0.39  0.03 | 0.45  0.02 | 0.38  0.03 |
| USA | ^36^ | 0.13 | 0.14 | 0.11 |
| BRA |  | 0.04 | 0.04 | 0.04 |
| ECU | ^6^ | 0.14 | 0.10 | 0.16 |
| CHN | ^47^ | 0.13 | 0.15 | 0.12 |
| Average, global north | | 0.06 | 0.07 | 0.08 |
| Average, global south | | 0.09 | 0.07 | 0.10 |
| Average, global | | 0.06 | 0.07 | 0.08 |

In the reference scenarios, the CO_2_ intensity of total energy consumption is expected to decline by 27% (on average) between 2020 and 2050—by 28% in the global north and 11% in the global south. If ambitious decarbonization measures are applied, the global average CO_2_ intensity of total energy consumption can be reduced by an additional 0.09 t CO_2_/MWh in 2050, i.e., by 70% compared to the 2020-level and by 58% compared to the RS level. Globally, the CO_2_ intensity in 2050 is expected to be 11% higher in the case of residential buildings than in the case of commercial buildings. The building sector in the global south is expected to have 43% higher CO_2_ intensity than in the global north.

## 3.2. Share of electricity in the total energy and heating demands of buildings

Supplementary Table 3.3. Share of electricity in the total energy and heating demands of buildings. Values for year 2020 are based on the baseline reported for each country, whereas the values for years 2030 and 2050 are based on the modeling scenarios.

| **Country** | **Of total energy demand  (%)** | | | **Of demand for space heating and hot water  (%)** | | |
| --- | --- | --- | --- | --- | --- | --- |
|  | **2020** | **2030** | **2050** | **2020** | **2030** | **2050** |
| AUT | 20% (av)  16% (min) 21% (max) | 23% (av)  17% (min) 27% (max) | 32% (av)  19% (min) 40% (max) | 8% (av)  8% (min)  9% (max) | 7% (av)  7% (min)  7% (max) | 8% (av)  6% (min) 8% (max) |
| BEL | 17% (av)  14% (min) 18% (max) | 20% (av)  14% (min) 23% (max) | 29% (av)  16% (min) 39% (max) | 7% (av)  7% (min) 7% (max) | 7% (av)  6% (min)  7% (max) | 14% (av) 6% (min)  19% (max) |
| BGR | 24% (av)  18% (min) 25% (max) | 27% (av)  18% (min) 31% (max) | 34% (av)  19% (min) 42% (max) | 17% (av)  17% (min) 17% (max) | 16% (av)  14% (min) 18% (max) | 13% (av)  11% (min)  18% (max) |
| CYP | 9% (av)  5% (min)  10% (max) | 11% (av)  6% (min) 12% (max) | 15% (av)  7% (min) 18% (max) | 16% (av)  16% (min) 16% (max) | 17% (av) 16% (min) 17% (max) | 17% (av)  16% (min)  19% (max) |
| CZE | 21% (av)  17% (min) 22% (max) | 24% (av)  17% (min) 28% (max) | 34% (av)  19% (min) 43% (max) | 9% (av)  9% (min)  9% (max) | 7% (av)  7% (min)  8% (max) | 11% (av)  7% (min)  15% (max) |
| DNK | 21% (av)  15% (min) 23% (max) | 24% (av)  15% (min) 28% (max) | 35% (av)  18% (min) 42% (max) | 7% (av)  7% (min)  7% (max) | 6% (av)  6% (min) 6% (max) | 5% (av)  4% (min) 6% (max) |
| EST | 22% (av)  17% (min) 23% (max) | 24% (av)  16% (min) 27% (max) | 29% (av)  18% (min) 34% (max) | 13% (av)  13% (min) 14% (max) | 9% (av) 8% (min)  9% (max) | 4% (av)  4% (min)  4% (max) |
| FIN | 27% (av)  24% (min) 29% (max) | 30% (av)  24% (min) 34% (max) | 37% (av)  26% (min) 46% (max) | 23% (av)  23% (min) 24% (max) | 22% (av)  21% (min) 24% (max) | 20% (av)  19% (min)  22% (max) |
| FRA | 24% (av)  17% (min) 25% (max) | 26% (av)  18% (min) 30% (max) | 40% (av)  21% (min) 49% (max) | 13% (av)  13% (min) 14% (max) | 11% (av)  11% (min) 12% (max) | 14% (av)  9% (min)  18% (max) |
| DEU | 21% (av)  17% (min) 23% (max) | 24% (av)  17% (min) 27% (max) | 35% (av)  19% (min) 46% (max) | 4% (av) 4% (min)  4% (max) | 4% (av)  4% (min)  4% (max) | 17% (av)  5% (min)  27% (max) |
| GRC | 21% (av)  15% (min) 23% (max) | 24% (av)  17% (min) 28% (max) | 34% (av)  19% (min) 40% (max) | 11% (av)  10% (min) 11% (max) | 10% (av)  10% (min) 11% (max) | 14% (av)  8% (min)  16% (max) |
| HUN | 21% (av)  17% (min) 22% (max) | 24% (av)  18% (min) 28% (max) | 32% (av)  20% (min) 39% (max) | 7% (av)  7% (min)  7% (max) | 7% (av)  6% (min)  7% (max) | 9% (av)  5% (min)  12% (max) |
| IRL | 22% (av)  19% (min) 23% (max) | 25% (av)  19% (min) 29% (max) | 35% (av) 21% (min) 42% (max) | 11% (av)  11% (min) 11% (max) | 10% (av)  10% (min) 11% (max) | 14% (av)  8% (min)  17% (max) |
| ITA | 23% (av)  18% (min) 24% (max) | 26% (av)  19% (min) 28% (max) | 37% (av)  22% (min) 43% (max) | 7% (av)  7% (min)  7% (max) | 7% (av)  6% (min) 8% (max) | 18% (av)  6% (min)  23% (max) |
| LVA | 12% (av)  8% (min)  13% (max) | 13% (av)  8% (min) 16% (max) | 16% (av)  8% (min) 19% (max) | 4% (av)  4% (min)  4% (max) | 3% (av)  3% (min)  3% (max) | 3% (av) 2% (min)  3% (max) |
| LTU | 16% (av)  12% (min) 18% (max) | 19% (av)  13% (min) 22% (max) | 28% (av)  15% (min) 33% (max) | 4% (av)  4% (min)  4% (max) | 4% (av)  4% (min)  5% (max) | 6% (av)  4% (min)  9% (max) |
| LUX | 28% (av)  23% (min) 30% (max) | 32% (av)  24% (min) 35% (max) | 40% (av)  26% (min) 46% (max) | 5% (av)  5% (min)  5% (max) | 5% (av)  5% (min)  6% (max) | 9% (av)  5% (min)  12% (max) |
| MLT | 25% (av)  16% (min) 28% (max) | 27% (av)  16% (min) 29% (max) | 34% (av)  18% (min) 40% (max) | 31% (av)  31% (min) 31% (max) | 24% (av)  24% (min) 25% (max) | 19% (av)  18% (min)  20% (max) |
| NLD | 16% (av)  11% (min) 17% (max) | 19% (av)  12% (min) 24% (max) | 30% (av)  15% (min) 42% (max) | 6% (av)  5% (min)  6% (max) | 5% (av)  5% (min) 6% (max) | 14% (av)  4% (min)  22% (max) |
| NOR | 85% | 85% | 85% (RS)  90% (DS) | 75% | 75% | 73% (rs)  74% (DS) |
| POL | 19% (av) 15% (min) 20% (max) | 23% (av)  15% (min) 27% (max) | 32% (av)  17% (min) 41% (max) | 5% (av)  5% (min)  5% (max) | 5% (av)  5% (min)  6% (max) | 13% (av)  5% (min) 17% (max) |
| PRT | 24% (av) 17% (min) 25% (max) | 27% (av)  18% (min) 31% (max) | 37% (av) 21% (min) 46% (max) | 17% (av)  17% (min) 17% (max) | 13% (av)  13% (min) 14% (max) | 11% (av)  11% (min)  12% (max) |
| ROU | 19% (av) 15% (min) 20% (max) | 22% (av)  17% (min) 25% (max) | 32% (av)  19% (min) 39% (max) | 3% (av)  3% (min)  3% (max) | 3% (av)  3% (min)  4% (max) | 6% (av)  5% (min) 9% (max) |
| SVK | 18% (av) 15% (min) 20% (max) | 22% (av)  15% (min) 25% (max) | 31% (av)  17% (min) 38% (max) | 5% (av)  5% (min) 5% (max) | 5% (av)  4% (min)  6% (max) | 9% (av)  4% (min)  13% (max) |
| SVN | 28% (av) 24% (min) 29% (max) | 32% (av)  25% (min) 35% (max) | 40% (av)  27% (min) 45% (max) | 9% (av)  8% (min)  9% (max) | 6% (av)  5% (min)  6% (max) | 5% (av)  3% (min)  6% (max) |
| ESP | 23% (av) 16% (min) 25% (max) | 26% (av)  17% (min) 30% (max) | 35% (av)  21% (min) 41% (max) | 10% (av)  10% (min) 10% (max) | 8% (av)  7% (min) 9% (max) | 15% (av)  9% (min)  19% (max) |
| SWE | 31% (av) 25% (min) 33% (max) | 35% (av)  26% (min) 39% (max) | 45% (av)  29% (min) 54% (max) | 20% (av)  19% (min) 20% (max) | 16% (av)  15% (min) 17% (max) | 13% (av)  10% (min)  16% (max) |
| GBR | 21% (av) 21% (min) 21% (max) | 22% (av) 20% (min) 24% (max) | 31% (av)  21% (min) 40% (max) | 8% (av)  8% (min)  9% (max) | 8% (av)  6% (min)  8% (max) | 17% (av) 4% (min)  28% (max) |
| USA** | 47% (RS) | 49% (RS) | 51% (RS) | 14% (RS) | 13% (RS) | 13% (RS) |
| BRA | 68% | 74% | 78% | 13% | 14% | 13% |
| ECU* | 34% | 35% (RS) 36% (DDP) | 40%(RS) 43% (DDP) | 11% (av) | 12% (av) | 12% (av) |
| CHN | 28% (Ref)  29% (TEP)  32% (Elec.) | 36% (Ref)  39% (TEP)  45% (Elec.) | 45% (Ref)  55% (TEP)  69% (Elec.) | 5% (Ref)  7% (TEP)  9% (Elec.) | 7% (Ref)  12% (TEP)  17% (Elec.) | 13% (Ref)  25% (TEP)  47% (Elec.) |
| *Average temperatures during the year remains reasonably comfortable, thus space heating is not an issue in Ecuador ^48,49^. Ecuador only reports electric water heating in residential buildings. ** The USA model, Scout, does not have these breakdowns for the DSs, so only the reference case is currently reported | | | | | | |

# Supplementary Notes/Methods 1. Comparative summary of national, sectoral and global models.

Supplementary Table 4 compares the scenarios developed in the global models and aimed at keeping the increase of global temperature below 1.5°C–2°C by 2050 (Grubler et al.^7^ Levesque et al. 2021^6^, SR 15C IPCC^9^, SDSN – IDDRI^41^, and IEA-ETP 2020^20^) to the results of the DSs obtained with our sectoral models. Broadly speaking, decarbonization measures in the considered global and sectoral scenarios aim to reduce FEC via energy-efficient management of new and existing buildings, and to increase energy supply from low emissive sources via electrification of the building’s energy demand, increased use of RESs on-site, and reduced carbon intensity of on-grid electricity. In all cases, these measures are to a certain extent counteracted by the growing energy demand in developing regions. Below, we compare how these key means of decarbonization of the building sector develop in the different models and target scenarios.

**Floor areas:** Both the global and sectoral models imply a certain increase in the energy demand in the coming decades: IEA-ETP^20^ estimates that worldwide, the floor area will more than double from 2020 reference in 2070; Grubler et al.^7^ in their low energy demand scenario meeting the 1.5°C target assume growth rates of floor area per capita of 26%–38%, implying a global increase of the total residential floor area by about 48% between 2020 and 2050, occurring mainly in the global south, while in the global north the residential floor space demand remains rather saturated; in the commercial sector, the total floor area is expected to increase by 68% globally. In our study, a more modest increase of 16%–18% is indicated between 2020 and 2050. However, both in our study and in that of Grubler et al.^7^, the global average floor area in the residential and commercial sector converges to around 30 m^2^ per capita in 2050, indicating the coherence of assumptions on the energy demand in the global and sectoral models. As we discuss in the main text, global provision of decent living standards is a key sufficiency measure implying a delicate balance between energy management and allocation of living spaces for which the operational feasibility and political implications are being further studied^21-24^.

**Renovation rates**: For all scenarios, renovation rates are higher in the global models than in the DSs of the sectorial models. In SDSN – IDDRI^41^, renovation rate is estimated at 2.3% per annum (2°C scenario). IEA-ETP^20^ estimates that around 53% of the remaining building stock will be renovated by 2050, with higher renovation rates in the global north than in the global south. Similarly, the results presented in Grubler et al.^7^ assume doubling retrofit rate by 2050 along with introducing stricter standards for new buildings (1.5°C scenario). These results can be compared to the lower annual renovation rates assumed in the sectoral models, with the global average value of 1.4% and varying from 1% to 2% depending on the region and sub-sector (R/C), which agrees with other literature finding that national roadmaps generally focus more on new and public buildings than existing buildings, despite the fact that the latter are naturally larger in number and total floor area, and perform less energy efficiently^12^.

**Electrification and decarbonization of the energy system:** Electrification is pivotal when it comes to decarbonizing the building sector under the assumption that RES electricity — wind and solar — will continue to grow in the coming years. Generally, RESs are accounted for differently depending on the capabilities of the models. For instance, most of the sectorial models allow to study on-site RES specifically (see Supplementary Table 2) but can only account for energy supply from low carbon fuels sources (e.g., nuclear energy, biomass, hydrogen) indirectly through the carbon intensity of electricity production. In turn, global models may typically consider the share of the individual fuel sources within the scenario calculations. With this in mind, in Table 2 below, we focus on the share of electricity from RES because it is a key indicator for which explicit information is provided in all the studies included. Share of electricity in the total energy demand in 2050 is estimated in the range of 42-82% in the global models. For the 1.5°C scenario, while Grubler et al.^7^ gives slightly higher values for the global north, the results in Levesque et al. 2021^6^ indicate significantly higher share of electricity in the global south (up to 82%) than in the global north (42%–61%). In our sectoral models, the global average share of electricity in the total energy demand in 2050 is estimated at 38%, and it is as well higher in the global south (62%, while the corresponding value for the global north is 35%). As noted above, the sectorial models consider energy sources beyond electricity differently depending on the national context and include both fossil sources and RES, such as bioenergy (e.g., NW Europe in the Inver/EE-Lab). Regarding electricity from RESs, the global models provide high values, i.e., in SR 15C IPCC^9^ the global average is assumed to reach up to 97% in 2050; in Grubler et al.^7^ and IEA-ETP 2020^20^ the numbers are 85% and 81%, respectively. Our sectoral studies assume significant differences between regions, i.e., 38% in the global north and 80% in the global south. Estimates for carbon intensity of electricity production in the global models for 2050 vary within the range 15–45 g CO_2_/kWh. In particular, SR 15C IPCC^9^ indicates that reaching the 2°C target would require carbon intensity of electricity production to be around 45 g CO_2_/kWh, while the 1.5°C target would require further reducing it to 30 g CO_2_/kWh. These estimates are significantly higher than values of carbon intensity of electricity production given in our study for RS – 133 g CO_2_/kWh globally, with regional differences: 260 g CO_2_/kWh in the global south, and 130 g CO_2_/kWh in the global north.

**Total CO_2_ emissions and emission reductions:** For the RS, adding up regional results from our sectoral models shows a global 18% CO_2_ emissions increase from 5.7 GtCO_2_ in 2020 to 6.7 GtCO_2_ in 2050. This is mainly driven by China, responsible of 70% of this increase. Per region, EU (both NW and SE) and the USA show reductions of 42% and 6%, respectively, while China and SA show increase of 44% and 32%. Conversely, DS leads to emission decrease of around two thirds of current levels by 2050, down to 1.9 GtCO_2_ (direct + indirect emissions). These total emissions can be compared to the results obtained in the global models: for the 2°C target, SR 15C IPCC^9^ estimates 2.1 GtCO_2_ in 2050, and SDSN – IDDRI^41^ 1.7 GtCO_2_ (both numbers concern direct emissions). For the 1.5°C target, SR 15C IPCC^9^ estimates 1.4 GtCO_2_ in 2050 (direct emissions), and Levesque et al. 2021^6^ 1.3 GtCO_2_ (direct + indirect emissions). Modelling results in Levesque et al. 2021^6^ indicate that decarbonization of energy supply accounts for 66% of the emission reductions in 2050, compared to the baseline, especially in the global south. In the intermediary scenario of IEA-ETP^20^, total emissions in 2050 at 1.7 GtCO_2_ are estimated, from which two thirds originate in the residential sector, and about 44% are indirect emissions originating from energy supply.

**Achievement of 2°C targets:** The results of the comparison of CO_2_ emissions and emission reductions in our sectoral models to those obtained in the global models indicate that the levels of ambition of the national developments represented in the DS would likely achieve the 2°C scenario goals. These are however achieved on a different balance of assumptions for floor area developments and renovations of existing buildings, both lower in sectoral models, which compensates their opposing effects. However, such comparison of the total emission levels and the implementation rates of mitigation measures also indicates that the national developments currently assumed in terms of rates renovation rates (1.4% on average in the sectoral DS), share of RESs (38% on average in the sectoral DS), share of electricity (38%–80% on average in the sectoral DS) and carbon intensity of electricity production may not be sufficient to reach the temperature goal of 1.5°C.

**Additional efforts in line with 1.5°C targets:** Beyond the efforts implied in the DS of our sectoral models, decarbonization scenarios in line with 1.5°C target would require additional efforts in terms of at least 1% higher renovation rate (to a global average of 2.4%), and 4%–14% larger share of electricity (to a global average of 70%). Energy production would need to be further decarbonized, implying a 3%–30% larger share of renewables (mostly in the global north) and carbon intensity of electricity production 4–6 times lower (to a global average of 30 g CO_2_/kWh). This implies that the national decarbonization plans that the DSs the sectoral models are meant to represent need to be made more ambitious and comprehensively include for both new and existing buildings all demand reductions, sufficiency, efficiency, electrification and decarbonization of energy production to maintain global warming below 1.5°C.

*Supplementary Table 4. Comparative summary of the key assumptions in decarbonization scenarios of the national sectoral and global models. Regions: GS represents the “global south”; GN, the “global north”; and W, “worldwide”. All countries included in our study belong to the GN except for Brazil and Ecuador that belong to GS.*

| **Reference** | **Region** | **Floor area (2050)** | **Renovation rates** | **Share of electricity in total energy demand (2050)** | **RES electricity**  **(2050)** | **Carbon intensity of electricity**  **(2050)** | **Total emissions (GtCO_2_) (2050)** |
| --- | --- | --- | --- | --- | --- | --- | --- |
| SR 15C IPCC  (1.5°C, 2°C) | W | - | N/A | 2°C: 50%  1.5°C: 60% | 2°C: -  1.5°C: 59–97% | 2°C: 45 g CO_2_/kWh^1^  1.5°C: 30 g CO_2_/ kWh^1^ | 2°C: 2.1 GtCO_2_^1^  1.5°C: 1.4 GtCO_2_^1^  SR15 scenario only reports direct CO_2_ under the assumption that emissions from electricity are nearly zero around 2050. |
| Grubler et al., 2018  (1.5°C) | GN | R: roughly constant at 30 m^2^/cap; C: +44% to 23 m^2^/cap | Doubling retrofit rate | 59%^2^ | 68%^3^ | - | - |
|  | GS | R: +32% to 29 m^2^/cap; C: +50% to 9 m^2^/cap | Standards for new buildings | 56%^4^ | 77%^5^ | - | - |
|  | W | R: +26% to 29 m^2^/cap; C: +38% to 11 m^2^/cap | - | 52%^6^ | 85%^7^ | - | - |
| IEA-ETP, 2020  (“net zero emissions by 2070” implying "below 1.8°C by 2100”) | GN | - | 64% of the building stock remaining by 2050^8^ | - | - | - | - |
|  | GS | Half of the floor area additions in sub-Saharan Africa and India | 41% of the building stock remaining by 2050^9^ | - | - | - | - |
|  | W | Absolute (not /cap) increases more than twofold to 2070 | 53% of the building stock remaining by 2050^10^ | 42%^11^ | 81%^12^ | 18 g CO_2_/kWh^13^  From 463 gCO_2_/kWh in 2019 to below zero in net terms around 2055. | R – total: 1.2 GtCO_2_ (81% reduction compared to 2019)^14^  R – direct: 0.7 GtCO_2_ (88% reduction compared to 2019)^14^  R – indirect: 0.5 GtCO_2_ (67% reduction compared to 2019)^14^  C – total: 0.5 GtCO_2_ (83% reduction compared to 2019)^14^  C – direct: 0.25 GtCO_2_ (89% reduction compared to 2019)^14^  C – indirect: 0.25 GtCO_2_ (67% reduction compared to 2019)^14^ |
| SDSN – IDDRI, 2015  (2°C) | GN | - | 2.3%^15^ | - | - | 15 g CO_2_/kWh ^16^ | - |
|  | GS | - | - | - | - | 22 g CO_2_/kWh ^17^ | - |
|  | W | - | - | 42%^18^ | - | 37 g CO_2_/kWh ^19^ | Direct: 1.7 GtCO_2_ (74% reduction compared to 2010)^20^ in 16 countries representing 74% of current global GHG emissions |
| Levesque et al, 2021  (1.5°C) | GN | As baseline | Building envelope upgrades and improved conversion efficiencies | 42–61% | - | - | Total: 0.6 GtCO_2_^22^  Indirect emissions account for 49% of emission reductions compared to baseline in 2050 |
|  | GS | As baseline |  | 65–82% | - | - | Total: 0.7 GtCO_2_^22^  Indirect emissions account for 73% of emission reductions compared to baseline in 2050 |
|  | W | As baseline |  | 75% | - | 10-20 g CO_2_/kWh^21^ | Total: 1.3 GtCO_2_ (86% reduction compared to 2015)^22^; Indirect emissions account for 66% of emission reductions compared to baseline in 2050 |
| This study | GN | EU–NW: R, + 4% increase between 2020 and 2050 (to 43 m^2^/cap in 2050); C, + 8% (to 15 m^2^/cap).  EU–SE: R, + 9% increase between 2020 and 2050 (to 32 m^2^/cap in 2050); C, + 15% (to 10 m^2^/cap).  CHN: R, + 36% increase between 2020 and 2050 (to 46 m^2^/cap in 2050); C, + 35% (to 17 m^2^/cap).  USA: R, + 15% increase between 2020 and 2050 (to 71 m^2^/cap in 2050); C, + 14% (to 30 m^2^/cap). | GN : 1.3%  EU–NW :  R, 1.2%–1.6%; C, 1.5%–1.8%.  EU–SE :  R, 1.3% ;  C, 1.5%.  CHN:  R, 2% ;  C, 2%.  USA:  R, 1%;  C, 1%. | GN: 35% | 38% | RS: 130 g CO_2_/kWh | GN (DS): 1.87 GtCO_2_  EU–NW (DS): 0.06 GtCO_2_  EU–SE (DS): 0.02 GtCO_2_  USA (DS): 0.76 GtCO_2_  CHN (DS): 1.02 GtCO_2_ |
|  | GS | SA: R, + 12% increase between 2020 and 2050 (to 20 m^2^/cap in 2050); C, + 19% (to 7 m^2^/cap). | 1.5% | 62% | 80% | RS: 260 g CO_2_/kWh | DS: 0.03 GtCO_2_ |
|  | W | R, + 16% increase between 2020 and 2050 (to 42 m^2^/cap in 2050); C, + 18% (to 16 m^2^/cap). | 1.4% | 38% | - | RS: 133 g CO_2_/kWh | DS: 1.9 GtCO_2_ |

^1^Visual from Figure 2.22. Carbon Emissions; Carbon Intensity.

^2^ Visual estimation from Figure 20, which includes all sectors.

^3^Visual estimation from Figure 20, which refers to the total final energy consumption.

^4^ Visual estimation from Figure 21, which includes all sectors.

^5^ Visual estimation from Figure 21, which refers to the total FEC.

^6^ Visual estimation from Figure 13, which refers to the final energy—electricity. Surprisingly, this value is not between the range of the values for GN and GS as the values are taken from different figures, as explained in footnotes 1 and 3.

^7^ Visual estimation from Figure 13, which refers to secondary energy [electricity] from hydro/wind/solar power.

^8^ Unweighted mean of 63% (NA), 64% (EU), and 64% (other advanced economies).

^9^ Unweighted mean of 41% (India), 52% (China) and 30% (other emerging economies).

^10^ Unweighted mean of 63% (NA), 64% (EU), 64% (other advanced), 41% (India), 52% (China) and 30% (other emerging).

^11^ Linear extrapolation from values for 2040 (30.1%) and 2070 (47.5%). Table 2.3 lists the energy for all sectors (not only buildings).

^12^ Percentage of power generation from Figure 3.2.

^13^ Visual estimation from Figure 3.2. Electricity only.

^14^Visual estimation from Figure 3.18.

^15^ Based on assumption of 600 000 buildings retrofitted per year in France (IDDRI) and 25 660 725 dwellings in metropolitan France in 2005 (Ribas Portella 2012).

^16^ Visual estimation from Figure 6. Australia, Canada, France, Germany, Italy, Japan, Korea, Russia, UK, USA. Electricity only.

^17^ Visual estimation from Figure 6. Brazil, China, India, Indonesia, Mexico, South Africa. Electricity only.

^18^ Visual estimation from Figure 7. Electricity and hydrogen (likely produced with electricity) share of final energy consumption.

^19^ Visual estimation from Figure 6. Australia, Brazil, Canada, China, France, Germany, India, Indonesia, Italy, Japan, Korea, Mexico, Russia, South Africa, UK, USA.

^20^Visual estimation from Figures 1 and 11.

^21^Visual estimation from Figure 7. Emission intensity of electricity, 1.5°C - EG scenario,

^22^Visual estimation and percentages from Figure 5. Baseline and 1.5°C - EG scenarios.

# Supplementary References 1. Numerical output per figure.

(Excel file attached)

# References

1. Gerhardt, N. et al. Hydrogen in the energy system of the future: Focus on heat in buildings. (2020).
2. Haley B, Kahrl F, Moore J, Jones AD, Torn MS, Mcjeon H. Deep Decarbonization Pathways Project (2015). Pathways to deep decarbonization 2015 report, SDSN - IDDRI.
3. Mantzos, L., Wisenthal, T., Neuwahl, F. & Rózsai, M. The POTEnCIA Central scenario: An EU energy outlook 2050. file:///S:/eftec_library/documents/214 - MEPF Socio-econ of Marine Aggs/SGRST-08-03 FINAL_REPORT_PENDING_ISBN_NUMBER including SGRST-08-01.pdf (2019) doi:10.2760/78212.
4. Standards Norway. NS 3720:2018 Metode for klimagassberegninger for bygninger (Method for greenhouse gas calculations for buildings). 40 (2018).
5. Lausselet, C., Borgnes, V. & Brattebø, H. LCA modelling for Zero Emission Neighbourhoods in early stage planning. Build. Environ. 149, 379-389 (2019).
6. Gibon, T., Arvesen, A. & Hertwich, E. G. Life cycle assessment demonstrates environmental co-benefits and trade-offs of low-carbon electricity supply options. Renew. Sustain. Energy Rev. 76, 1283-1290 (2017).
7. Bataille, C. et al. Net-zero deep decarbonization pathways in Latin America: Challenges and opportunities. Energy Strateg. Rev. 30, (2020).
8. Shen, X. & Liu, B. Changes in the timing, length and heating degree-days of the heating season in central heating zone of China. Sci. Rep. 6, 1-10 (2016).
9. Miño-Rodríguez, I., Naranjo-Mendoza, C. & Korolija, I. Thermal Assessment of Low-Cost Rural Housing-A Case Study in the Ecuadorian Andes. Buildings 6, 36 (2016).
10. Schweiker, M. et al. The Scales Project, a cross-national dataset on the interpretation of thermal perception scales. Sci. Data 6, 289 (2019).
