# Supplementary material for: A global comparison of building decarbonization scenarios by 2050 towards 1.5–2 °C targets
Source: Nat Commun. 2022 Jun 2;13:3077. doi: 10.1038/s41467-022-29890-5 (PMC9163154; doi:10.1038/s41467-022-29890-5)
Supplement: Supplementary file 3 — Source Data [file 41467_2022_29890_MOESM3_ESM.pdf]

nature portfolio

Peer Review File

A global comparison of building decarbonization scenarios by 2050 towards 1.5 -2 °C targets

Open Access This file is licensed under a Creative Commons Attribution 4.0 International License, which permits use, sharing, adaptation, distribution and reproduction in any medium or format, as long as you give appropriate credit to the original author(s) and the source, provide a link to the Creative Commons license, and indicate if changes were made. In the cases where the authors are anonymous, such as is the case for the reports of anonymous peer reviewers, author attribution should be to 'Anonymous Referee' followed by a clear attribution to the source work. The images or other third party material in this file are included in the article's Creative Commons license, unless indicated otherwise in a credit line to the material. If material is not included in the article's Creative Commons license and your intended use is not permitted by statutory regulation or exceeds the permitted use, you will need to obtain permission directly from the copyright holder. To view a copy of this license, visit http://creativecommons.org/licenses/by/4.0/.

Reviewer comments, first round

Reviewer #1 (Remarks to the Author):

Overall comments on the paper

As noted by the authors, decarbonising the buildings sector is of critical importance to meeting climate change targets. The large array of sector-specific models utilised in this paper to examine how this can be done is therefore of potentially great value to understanding the technologies and measures, and how they interact, to produce low-carbon pathways in the buildings sector.

However, as it stands, this paper fails to make a compelling contribution to this question, simply because most of its findings sections read like a long list of results from the models which are implementing pre-determined Ambitious Decarbonisation Scenarios (which, in the context of the need to keep to the Paris Agreement goal of 1.5C, are not really that ambitious). No really striking insights are highlighted that give a feel for what is driving these model results, nor what their real-world relevance is with respect to competing decarbonisation priorities and costs, either when comparing the building sectors to other sectors (e.g. electricity decarbonisation) or when comparing different building sector options (e.g. prosumer choices versus electrification of heat versus energy efficiency).

There is also a lack of compelling insight beyond findings that have already been widely discussed in terms of decarbonising buildings. We know energy efficiency and decarbonisation of energy are critical (in the buildings sectors as well as other sectors), so what does this analysis add that's new, or that other analyses have failed to capture? What's the big finding? This really isn't immediately obvious amongst the mass of numbers presented.

Of critical importance is the lack of reflection on the key finding presented in the abstract – that emissions reductions of 20-40% below the RS result from the ADS. We are in an international context that requires close to zero emissions from all sectors by 2050, if we are to keep on track to meet the goal of the Paris Agreement, given concerns over the large-scale deployment of carbon removal technologies. Furthermore, there are high-profile, illustrative scenarios ("P1" in the IPCC's Special Report on Global Warming of 1.5oC, which is based on the Gruebler et al., 2018, Low Energy Demand paper cited in this study) that achieve close to zero emissions in all sectors (including buildings). Is this study therefore categorically claiming that P1 is impossible? If so, what is the credibility of the underlying assumptions and modelling methods used in this paper that demonstrates such a striking result? There is little in the paper that allows a critical assessment of other scenarios which achieve a much deeper decarbonisation of the buildings sector.

Additionally, is this really the most comprehensive buildings study ever conducted? Yes, it employs several models which are sector-specific and in aggregate cover a large share of the world's emissions. However, the IEA's Energy Technology Perspectives (ETP) analysis has for several years examined each energy sector (including buildings) in detail, combining bottom-up models of buildings with energy system modelling based on the TIMES framework. The ETP analysis covers every world country / region, and the level of detail provided in ETP reports (at least those before 2015, which had detailed country profiles) is relatively high compared to the level of detail in this paper. Additionally, studies such as the UN SDSN Deep Decarbonisation Pathways Project use a range of technology-rich, detailed TIMES / MARKAL models to examine the sectoral decarbonisation patterns in most major regions. Many of these models have separate commercial and residential buildings sectors. See:
https://www.iddri.org/sites/default/files/import/publications/ddpp_2015synthetisreport.pdf

As such, I can't recommend this for publication in Nature Communications. I think that, with careful revision to the text, so that it presents its results in a much more contextualised manner, explaining what drives them and how they compare to other building decarbonisation pathway findings, and how / why they are more credible as a result of their sectoral detail, the paper could

be suited to a more specialised, technical publication.

Specific comments on the text

P2, Line 39: I would expect to see an authoritative energy and emissions data source (e.g. IEA, EDGAR, PRIMAP, CEDS) referenced for these facts, rather than another paper.

P2, Line 40: I'm sure there's a huge range of energy growth projections from the buildings sector. One only need look at reference scenarios from the SSP database and select final energy use in buildings to get this range. Simply quoting one figure from two sources (and do References 2 and 3 agree on 80% in a no policy scenario?) gives a sense of false precision.

P2, Line 43: Again, only one figure for required emissions reductions is quoted, and from one source (incidentally, Ref 1 is the same as Ref 4). There are many pathways to 2C, which no doubt have a range of emissions levels in 2050.

P2, Line 48: What about the potentially critical role of hydrogen in heating? Hydrogen boilers are already being mass manufactured by some companies. Are refs 5 and 6 representative of the span of literature that assesses the full range of low-carbon buildings solutions? I am not convinced they are, judging by the lack of comprehensive coverage of the range of solutions. The IPCC reports, or sources like IEA's roadmaps / energy technology perspectives, should be referenced (and indeed read) in order to cover the span of technologies.

P2, Line 55: Are the authors sure that the global models apply the same costs and technologies to the residential and commercial buildings sectors? Models like the TIMES Integrated Assessment Model (TIAM) have separate representation of these buildings sectors, with distinct technology specification possibilities.

P5, Line 104. Most of the discussion in the section "Ambitious decarbonisation scenarios versus reference scenarios" read like a technical annex to a report. I found there was very little discussion about why models and scenarios assumed certain technology trends or fuel use patterns, beyond simply stating that this is what they did. I think this comes down to a question of why the ADS scenarios for each region detailed in Suppl Mat 2 are useful and credible representations of what constitutes genuinely ambitious action. Take P6, line 128 as an example: is a 3% refurbishment rate realistically the most ambitious rate in Germany? What would be the costs of this relative to other low-carbon solutions like heating electrification? How is this choice resolved in the CoreBee model's optimisation? Or take P7, Line 146: Is the assumed rate of decarbonisation of electricity a credible maximum? How ambitious is the ADS assumption here?

P6, Fig 2: I got thoroughly confused when looking at the charts and legends in this figure. I didn't understand what Bas and Sce meant, why each region didn't have a clearly labelled RS and ADS scenario, and why some regions (EU and SA) had mean / min / max / Q1 / Q3 lines and other regions didn't. I also found the inclusion of both EU sub-regions and the US and SA regions on single panels really cluttered up the charts. It was as a result difficult to discern any meaningful patterns or trends in this figure.

P9, Line 243: Here there is an example of actually comparing some of the underlying dynamics in this modelling exercise with other studies i.e. the difference in floor area assumptions. How credible are those other studies with minimum sufficient living floor space? Is such a strikingly low floor space a key factor that drives those studies that get to much lower emissions reductions in 2050 compared to this study? This is the kind of contextualisation that would add credibility to the findings presented here.

P10, Figure 4: There's very little discussion in the text about what these 6 panels are showing, and it's quite hard to interpret any inter-regional differences or similarities, and implications, of this figure.

P10, Conclusions: The fact that the ADS scenarios don't go far enough to 2C begs the question of how ambitious these scenarios are, and whether they are insufficient by design, or whether there

are hard realities that mean buildings can't decarbonise further and faster. Some discussion on this point, and further justification / explanation of why these ADS scenarios are a useful guide to the realistic rate of decarbonisation, would be helpful.

P11, Line 273: I was surprised to see floor area as an outcome variable. Why isn't this an input assumption that drives model results, for example as part of the building structure input described in line 276? Or is it a result of GDP per capita assumptions?

Reviewer #2 (Remarks to the Author):

This is a collaborative study conducted by 20 researchers from 9 institutes. It summarizes various building energy stocking models and employs them to forecast energy demand and carbon emission of building sector to 2050. 32 countries across 4 continents are included in the study, including both developed and developing countries and areas. Detailed calculations and reasonable assumptions draw profound and valuable conclusions, which could be good reference for the researchers and policy makers around the world. As my point of view, it is the most comprehensive and in-depth study in the topic of carbon neutrality in buildings. The findings of this paper could be of interest not only for the building engineering but also for the others, like social, economy, etc.
One suggestion to improve the paper: add discussion before conclusion, about the limitation of the study and future work after this study.

Reviewer #3 (Remarks to the Author):

This manuscript is an international effort to test whether rapid decarbonization of the buildings sector can be achieved. This is a kind of ensemble piece, where model results from different regions are being compiled together. This has major implications for interpretation and I leave some suggestions below, along with several minor comments.

- Line 40, pls specify if this estimate is primary or final energy use, and whether it is electricity only or also fuels.
- Line 43, is the 32 Gt CO_2 specific to buildings? Either way, please note, as the introduction focuses solely on buildings.
- Lines 50-52, I don't disagree with this, but behavior does not feature prominently in the analysis, and there is no inclusion of rebound, so it may make sense to remove this sentence to improve narrative flow. Also, 'excessive lifestyles' is vague – how to define excessive? The paper does not set a minimum level of energy services to compare against.
- Line 65, unclear if this is 60% of global emissions or 60% of global building emissions.
- Table 1 has overlapping models for some countries in Europe – how were these multiple results handled?
- Status Quo section, can this also be broken up by building sector? The authors have argued that the sectorial representation in the modeling is one of the major advantages of their approach.
- Line 89, it does not appear that statistical correlation was done, might be better to avoid this term. Or better yet, perform statistical tests to test these hypotheses.
- Figure 1, is it also possible to see the mix of energy carriers, or perhaps the carbon intensity of average final energy use? This would help in interpreting the carbon emissions results. This is seen later in Figure 4.
- Figure 2, I assume NR in the graphs is 'non-residential'? Please change to C in order to match the caption and the rest of the text. It would help readers to note in the caption that Commercial is the dotted lines, Residential is the solid lines. The thick bars in the bar charts are the RS results. Honestly it is really hard to differentiate the lines. Authors could consider splitting up the graphs to show the two sectors separately, or by the two scenarios, or expand the color palette. Also, it looks from the legend like only Commercial is modeled for SA, but the bars show R and C results?
- Line 161, this text is hard to interpret: 'Total emissions under this scenario are 645 Mt CO_2 in 2050, compared with only 29% lower in the modelled RS (AEO2019-Ref).' Perhaps the % decrease that corresponds to the 645 number could be added so that readers can make a parallel

comparison?
- The time periods presented in the text do not always match. Figure 2 shows 2020-2050. The methods state that the start year is 2020, but the discussion of China's results make comparisons between 2015 and 2050. A minor related point: the supplement lists GDP in 2005 USD - presumably these are 2020 GDP numbers, why state in 2005 dollars?
- Is there meant to be a parallel between Figures 1 and 3? Some of the panels are different. There seems to be some overlap with Figure 4 as well – is this supposed to show only relative changes, while the comparison of Figures 1 and 3 shows absolute results? Perhaps there is a clever way to combine some of this information, or also show the RS results in Figure 3. Otherwise, I found myself constantly flipping back to earlier graphs to make comparisons.
- Socio-economic insights section, the 8100 kWh/cap result is duplicated in this paragraph. Also, it looks like there is a typo, it should be 100 m2/cap, not 1000.
- Figure 4, the connecting lines in the lower-right panel are a nice visual aid – I would recommend including for all of the panels.
- It doesn't appear that future climate change is accounted for here – this should be noted somewhere.
- I wish the results had more explanatory power. It isn't clear why what is driving the differences in results, and the discussion doesn't take full advantage of the bottom-up modeling framework. It would be good to highlight exactly what knowledge was gained here, as distinct from insights that are possible from integrated assessment modeling. For example, Figure 4 upper-right panel shows building energy demand vs carbon intensity, but it would be much more interesting to see emissions vs carbon intensity. Very few physical descriptors of the actual building stock are presented, really only floorspace. It would be good to see average U-values for example, or penetration of certain technologies.
- How do these results compare with those derived from national or international energy models? Do the bottom-up results corroborate existing aggregated results, or do they reveal patterns that were not evident previously?
- My last comment is the most general: it is hard to get a clear picture of the results because the models and scenarios being employed are different. This appears to be the strategy of the authors and there is no getting around it as each bottom up model was constructed independently. But, putting the results together side-by-side requires more discussion of proper interpretation for readers. The authors have done a nice job summarizing each model in the methods section, giving high-level information in Table 1, and presenting details in the Supplement. I would highly recommend some additional text to discuss common features across models. It should be clarified if all models are covering the same suite of energy end uses and technology options. It should be clarified if all models are considering the same suite technology options. For example, do all models consider appliance efficiency upgrades, or ground-source heat pump conversion, or is the technology suite considered country-dependent? Are new buildings built to different codes in each model, and what is the influence of new construction vs retrofitting existing buildings in each region? And for the ADS scenarios, if the actual background assumptions are completely different, how are readers supposed to interpret the results? If Europe decarbonizes its buildings more rapidly than the US and China, is this because the scenarios are not equivalent, more than the effect of population or status of the current building stock? Perhaps more quantitative information about the scenarios can be added to Table 1. In the end, if in fact the models and the scenarios are completely different and there is no fair way to compare results across countries, then the authors should be very careful in their discussions to caution readers and to explain that the results simply represent the most ambitious scenarios being considered in each country, and that even the formulation of the scenarios is a reflection of what national modelers believe is achievable.

Point-by-point response to the reviewers' comments

Reviewer #1

Overall comments on the paper

As noted by the authors, decarbonising the buildings sector is of critical importance to meeting climate change targets. The large array of sector-specific models utilised in this paper to examine how this can be done is therefore of potentially great value to understanding the technologies and measures, and how they interact, to produce low-carbon pathways in the buildings sector.

[Comment #1.1]

However, as it stands, this paper fails to make a compelling contribution to this question, simply because most of its findings sections read like a long list of results from the models, which are implementing pre-determined Ambitious Decarbonisation Scenarios (which, in the context of the need to keep to the Paris Agreement goal of 1.5C, are not that ambitious). No really striking insights are highlighted that give a feel for what is driving these model results, nor what their real-world relevance is with respect to competing decarbonisation priorities and costs, either when comparing the building sectors to other sectors (e.g. electricity decarbonisation) or when comparing different building sector options (e.g. prosumer choices versus electrification of heat versus energy efficiency).

Answer: We appreciate the reviewer´s concern and we have amended the manuscript accordingly. First, we have now made it clear in the beginning of the paper that the suit of measures is country-dependent based on the interventions deemed as viable by each modeller and removed the word "Ambitious" from the scenario definition, so that we now have "Decarbonisations Scenarios (DS)" instead of "Ambitious Decarbonisations Scenarios (DS)". Second, we have introduced new text to highlight key insights from the results of the modelling. In parallel, to better describe what is driving each model, we have included additional information on technology and demand developments for each of the models in Supplementary Material 2. References have been included to support all data and assumptions.

[Comment #1.2]

There is also a lack of compelling insight beyond findings that have already been widely discussed in terms of decarbonising buildings. We know energy efficiency and decarbonisation of energy are critical (in the buildings sectors as well as other sectors), so what does this analysis add that's new, or that other analyses have failed to capture? What's the big finding? This really isn't immediately obvious amongst the mass of numbers presented.

Answer: We understand the reviewer's point and see this is as an additional key remark. We have added a more detailed comparison of our results to those of global models, identifying exactly what drives the difference, both quantitatively and narratively. This comparison in

shown in a new Supplementary Material 5, and the insights from the comparison are presented through the paper, starting in the introduction.

[Comment #1.3]

Of critical importance is the lack of reflection on the key finding presented in the abstract – that emissions reductions of 20-40% below the RS result from the DS. We are in an international context that requires close to zero emissions from all sectors by 2050, if we are to keep on track to meet the goal of the Paris Agreement, given concerns over the large-scale deployment of carbon removal technologies. Furthermore, there are high-profile, illustrative scenarios ("P1" in the IPCC's Special Report on Global Warming of 1.5 oC, which is based on the Gruebler et al., 2018, Low Energy Demand paper cited in this study) that achieve close to zero emissions in all sectors (including buildings). Is this study therefore categorically claiming that P1 is impossible? If so, what is the credibility of the underlying assumptions and modelling methods used in this paper that demonstrates such a striking result? There is little in the paper that allows a critical assessment of other scenarios, which achieve a much deeper decarbonisation of the buildings sector.

Answer: We have addressed this concern in the revision throughout the whole storyline of the manuscript. The new comparison, described above in our reply to comment #1.2, includes Grubler et al, 2018 and other key global studies. Furthermore, for each of the modelling results, we have asked: "Can the DS attain carbon neutrality? If not, how could carbon neutrality be achieved?". The results from these queries for each of the models has been added in SM2.2, and overarching conclusions incorporated to the abstract and conclusions.

[Comment #1.4]

Additionally, is this really the most comprehensive buildings study ever conducted? Yes, it employs several models which are sector-specific and in aggregate cover a large share of the world's emissions. However, the IEA's Energy Technology Perspectives (ETP) analysis has for several years examined each energy sector (including buildings) in detail, combining bottom-up models of buildings with energy system modelling based on the TIMES framework. The ETP analysis covers every world country / region, and the level of detail provided in ETP reports (at least those before 2015, which had detailed country profiles) is relatively high compared to the level of detail in this paper. Additionally, studies such as the UN SDSN Deep Decarbonisation Pathways Project use a range of technology-rich, detailed TIMES / MARKAL models to examine the sectoral decarbonisation patterns in most major regions. Many of these models have separate commercial and residential buildings sectors. See: https://www.iddri.org/sites/default/files/import/publications/ddpp_2015synthetisreport.pdf

As such, I can't recommend this for publication in Nature Communications. I think that, with careful revision to the text, so that it presents its results in a much more contextualised manner, explaining what drives them and how they compare to other building decarbonisation pathway findings, and how / why they are more credible as a result of their sectoral detail, the paper could be suited to a more specialised, technical publication.

Answer: We have rephrased the aim and scope of the paper to address this comment, emphasizing that our work is a compilation of bottom-up studies. Furthermore, we have included substantial additional information from other references, including the ETP and IDDRI

reports, as well as the work by Levesque et al (2021) which, notably, already presents a comparison of modelling scenarios including the ETP reports as well as results from all models of the SSP database (see new Supplementary Material 5). We agree with the reviewer that ETP and EDDRI reports, and SSP database probably contain, among the tremendous amount of information included in the datasets available on demand, many of the specific information that our compiled studies provide. We believe that this should not undermine our effort, as there are various notable risks with these assessments. First, their comprehensiveness could make them inaccessible for the public, precisely as the reviewer is pointing in the following comment #1.5; in our study, we have tried to summarize and simplify the findings, while providing a level of disaggregation beyond the results presented in global assessments. Second, there is a risk that the assessments use similar core high-level data, for instance from the IEA, which lead to limited variations and insights from their results. Third, the nature of the models allows for massive uptake of solutions with uncertain large-scale implantation and high risks, such as BECCS, or, as we end up identifying in our paper, electrification of buildings' energy demand. In relation to the second and third points, in our study, we compile more feasible and expert validated assessments based on national data. We therefore believe that there is a value in validating with other modelling approached the results of global models, even if similar conclusions were reached, especially given the size of investments and the socio-technical transformations that the climate mitigation strategies in line with 1.5 and 2°C call for.

Specific comments on the text

[Comment #1.5]

P2, Line 39: I would expect to see an authoritative energy and emissions data source (e.g. IEA, EDGAR, PRIMAP, CEDS) referenced for these facts, rather than another paper.

Answer: We exchanged the reference by the IEA data in "Tracking Buildings 2020".

[Comment #1.6]

P2, Line 40: I'm sure there's a huge range of energy growth projections from the buildings sector. One only need look at reference scenarios from the SSP database and select final energy use in buildings to get this range. Simply quoting one figure from two sources (and do References 2 and 3 agree on 80% in a no policy scenario?) gives a sense of false precision.

Answer: We have now updated the sentence with the values from the most updated SSP database. As the number of references is limited in Nature Communications, we have extracted these values from several of the references that we are using. Besides, Riahi et al 2017 reports results from 5 models and scenarios, providing a solid summary of existing projections.

[Comment #1.7]

P2, Line 43: Again, only one figure for required emissions reductions is quoted, and from one source (incidentally, Ref 1 is the same as Ref 4). There are many pathways to 2C, which no doubt have a range of emissions levels in 2050.

Answer: Thanks for noticing the repetition of Ref 1-4, which we have corrected. We have additionally updated the sentence with a wider range of values of emission reductions from different scenarios and new references. As in the previous comment, due to the number of

references being limited in Nature Communications, we have extracted these values from several of the references that were already used.

[Comment #1.8]

Line 48: What about the potentially critical role of hydrogen in heating? Hydrogen boilers are already being mass manufactured by some companies. Are refs 5 and 6 representative of the span of literature that assesses the full range of low-carbon buildings solutions? I am not convinced they are, judging by the lack of comprehensive coverage of the range of solutions. The IPCC reports, or sources like IEA's roadmaps / energy technology perspectives, should be referenced (and indeed read) in order to cover the span of technologies.

Answer: The studies we have consulted addressing the role of hydrogen in the building sector agree that hydrogen is not a viable option when it comes to heating buildings. The amount of green electricity needed to produce green hydrogen for this purpose is 500 to 600 percent greater than the amount needed to power an equivalent number of heat pumps (Gerhardt, 2020). Hydrogen is only considered of relevance for the industry or transport sectors (EDDRI, 2015). Due to the word restriction, we have included a sentence as a foot of the caption of SM2.1, to justify with this is not contemplated in the "Technology and demand developments", including the reference to the study. As for the reviewers' concern, the span of considered literature, including additional recent references, assesses the full range of low-carbon buildings solutions.

[Comment #1.9]

Line 55: Are the authors sure that the global models apply the same costs and technologies to the residential and commercial buildings sectors? Models like the TIMES Integrated Assessment Model (TIAM) have separate representation of these buildings sectors, with distinct technology specification possibilities.

Answer: It was just an example. Some models may allow for separate input, but in reality, have the same values in both sectorial inputs. In any case, in the updated version of the manuscript, we decided to erase this sentence due to space limitations.

[Comment #1.10]

P5, Line 104. Most of the discussion in the section "Ambitious decarbonisation scenarios versus reference scenarios" read like a technical annex to a report. I found there was very little discussion about why models and scenarios assumed certain technology trends or fuel use patterns, beyond simply stating that this is what they did. I think this comes down to a question of why the ADS scenarios for each region detailed in Suppl Mat 2 are useful and credible representations of what constitutes genuinely ambitious action. Take P6, line 128 as an example: is a 3% refurbishment rate realistically the most ambitious rate in Germany? What would be the costs of this relative to other low-carbon solutions like heating electrification? How is this choice resolved in the CoreBee model's optimisation? Or take P7, Line 146: Is the assumed rate of decarbonisation of electricity a credible maximum? How ambitious is the ADS assumption here?

Answer: We have addressed this comment in a number of ways. First, we have included further information on the limitations of the DS (we now have “Decarbonisations Scenarios (DS)” instead of “Ambitious Decarbonisations Scenarios (DS)” see reply to comment #1.1). Second, we have included additional explanations along with references in each of the assumptions behind the scenarios in SM2.1. It is important to note that each model is conceived differently. Taking reviewer’ example, Corebee assumes the EU desirable renovation rates as input to calculate cost optimal solutions. Therefore, the level of renovation is an input parameter to the analysis. The ambition of such number (3%), which have been largely discussed in several policy outputs, may not be sufficient to achieve carbon neutrality, but it is the highest feasible rate based on the status of the sector in Europe. In terms of costs for the various technology developments, we understand and support the reviewer´s point. The costs/investments are, however, something that we decided to keep out of the scope of the study as it will require a larger analysis, thus, making difficult to keep the analysis concise. Please note that due to the journal´s text limitation (5,000 words), for the body of the text explanations were shortened to the essentials and further information was sent to the Supplementary Materials.

[Comment #1.11]

P6, Fig 2: I got thoroughly confused when looking at the charts and legends in this figure. I didn’t understand what Bas and Sce meant, why each region didn’t have a clearly labelled RS and DS scenario, and why some regions (EU and SA) had mean / min / max / Q1 / Q3 lines and other regions didn’t. I also found the inclusion of both EU sub-regions and the US and SA regions on single panels really cluttered up the charts. It was as a result difficult to discern any meaningful patterns or trends in this figure.

Answer: Thank you for highlighting this matter. We have worked on Fig. 2 to make it as easy as possible to read without removing any data. We have also revised the legend and axis titles in order to make them consistent throughout the paper. Additional explanation has also been included to highlight significant patterns and trends.

[Comment #1.12]

P9, Line 243: Here there is an example of actually comparing some of the underlying dynamics in this modelling exercise with other studies i.e. the difference in floor area assumptions. How credible are those other studies with minimum sufficient living floor space? Is such a strikingly low floor space a key factor that drives those studies that get to much lower emissions reductions in 2050 compared to this study? This is the kind of contextualisation that would add credibility to the findings presented here.

Answer: We have updated the section with more detailed comments and references to additional works on the impact of household sizes.

[Comment #1.13]

P10, Figure 4: There’s very little discussion in the text about what these 6 panels are showing, and it’s quite hard to interpret any inter-regional differences or similarities, and implications, of this figure.

Answer: We have addressed this point in three ways. First, we have improved the graph by including lines to link the data to make regional trends easy to follow. Whenever the results

allowed, we have also made the Y-axis start in 100% in order to make it easier to compare across regions. Also, we have included further information in the caption on how it could be read. In parallel, the bottom-right subplot of the figure (average emissions per capita versus average building energy demand) has been replaced by "average emissions per capita versus carbon intensity of energy demand" in order to better convey indicators across regions. All of this has been complemented with additional explanation on the results' section.

[Comment #1.14]

P10, Conclusions: The fact that the DS scenarios don't go far enough to 2C begs the question of how ambitious these scenarios are, and whether they are insufficient by design, or whether there are hard realities that mean buildings can't decarbonise further and faster. Some discussion on this point, and further justification / explanation of why these DS scenarios are a useful guide to the realistic rate of decarbonisation, would be helpful.

Answer: As highlighted in our response to Comment #1,1, to ensure that readers are aware that the results simply represent the most ambitious scenarios being considered in each country, and that the formulation of the scenarios is based on a reflection of what national modellers believe is achievable, explanations have been added throughout the storyline in the introductory, discussion, and method sections. We have further complemented this with a table in the Supplementary Material to explicitly address this matter (See SM2.2).

[Comment #1.15]

P11, Line 273: I was surprised to see floor area as an outcome variable. Why isn't this an input assumption that drives model results, for example as part of the building structure input described in line 276? Or is it a result of GDP per capita assumptions?

Answer: The floor area is a structural element of the models, thus in can be both an input and an output. To better illustrate this, the floor area as an input for the RS in 2020 is included in SM1. Likewise, the floor area as an output is now added in the title of the sixth column "Demographic and floor area development" in SM2. In line 276 we were describing the status quo in 2020, therefore, the floor area that refers to SM1 data.

Reviewer #2

[Comment #2.1]

This is a collaborative study conducted by 20 researchers from 9 institutes. It summarizes various building energy stocking models and employs them to forecast energy demand and carbon emission of building sector to 2050. 32 countries across 4 continents are included in the study, including both developed and developing countries and areas. Detailed calculations and reasonable assumptions draw profound and valuable conclusions, which could be good reference for the researchers and policy makers around the world. As my point of view, it is the most comprehensive and in-depth study in the topic of carbon neutrality in buildings. The findings of this paper could be of interest not only for the building engineering but also for the

others, like social, economy, etc. One suggestion to improve the paper: add discussion before conclusion, about the limitation of the study and future work after this study.

Answer: We sincerely appreciate the positive feedback from Reviewer#2. With respect to the suggestion of adding a discussion section, the limited number of headings in the template of this journal doesn't allow for a new section. We have, however, added text to make clear he limitations of our study, and provided complementary information (in Supplementary material 2.1 and 2.2) about key assumptions and drivers for each model in the study. Last, we have now clearly identified further work in the conclusions.

Reviewer #3

This manuscript is an international effort to test whether rapid decarbonization of the buildings sector can be achieved. This is a kind of ensemble piece, where model results from different regions are being compiled together. This has major implications for interpretation and I leave some suggestions below, along with several minor comments.

[Comment #3.1]

Line 40, pls specify if this estimate is primary or final energy use, and whether it is electricity only or also fuels.

Answer: It is primary energy consumption including electricity and fossil fuels. We have included additional text in the manuscript to clarify.

[Comment #3.2]

Line 43, is the 32 Gt CO2 specific to buildings? Either way, please note, as the introduction focuses solely on buildings.

Answer: It is Gt CO2 from building-related sources. However, to address the concerns by other reviewers we are now providing a wider range of values from different models, as well as the emissions reductions in terms of percentages instead of absolute values.

[Comment #3.3]

Lines 50-52, I don't disagree with this, but behavior does not feature prominently in the analysis, and there is no inclusion of rebound, so it may make sense to remove this sentence to improve narrative flow. Also, 'excessive lifestyles' is vague – how to define excessive? The paper does not set a minimum level of energy services to compare against.

Answer: We have accepted the reviewer´s proposal of deleting this sentence, as the issue is covered later in the paper under section "Socio-economic insights …".

[Comment #3.4]

Line 65, unclear if this is 60% of global emissions or 60% of global building emissions.

Answer: We have clarified in the text making clear it refers to 60% of global building emissions.

[Comment #3.5]

Table 1 has overlapping models for some countries in Europe – how were these multiple results handled?

Answer: We have added additional information in the method section to describe this, namely: "Some countries in Europe are addressed by more than one model, in which case the mean of the results per county has been calculated. All results are presented and conferred.".

[Comment #3.6]

Status Quo section, can this also be broken up by building sector? The authors have argued that the sectorial representation in the modeling is one of the major advantages of their approach.

Answer: We have improved the resolution of Figure 1 by disaggregating energy consumption and CO_2 emissions for residential and commercial buildings. Subsequently, the text describing the figures now collects the distinction between the residential and commercial subsectors.

[Comment #3.7]

Line 89, it does not appear that statistical correlation was done, might be better to avoid this term. Or better yet, perform statistical tests to test these hypotheses.

Answer: Indeed, we have not done a statistical analysis in this paper, so we have rephrased the text by linking to a review that supports the statement.

[Comment #3.8]

Figure 1, is it also possible to see the mix of energy carriers, or perhaps the carbon intensity of average final energy use? This would help in interpreting the carbon emissions results. This is seen later in Figure 4.

Answer: This information can be accessed in SM3 as an additional. The information on the carbon intensity is implicitly addressed in Figures 1 and 4 but we did not include an additional box as they already depict a lot of information and we didn't want to make them too complex for the reader.

[Comment #3.9]

Figure 2, I assume NR in the graphs is 'non-residential'? Please change to C in order to match the caption and the rest of the text. It would help readers to note in the caption that Commercial is the dotted lines, Residential is the solid lines. The thick bars in the bar charts are the RS results. Honestly it is really hard to differentiate the lines. Authors could consider splitting up the graphs to show the two sectors separately, or by the two scenarios, or expand the color palette. Also, it looks from the legend like only Commercial is modeled for SA, but the bars show R and C results?

Answer: We have updated the figures to consistently represent "R" for residential and "C" for commercial and have split the graphs to show the two sectors separately, as suggested. We have also worked on Fig. 2 to make it as easy as possible to read without removing any data.

[Comment #3.10]

Line 161, this text is hard to interpret: 'Total emissions under this scenario are 645 Mt CO2 in 2050, compared with only 29% lower in the modelled RS (AEO2019-Ref).' Perhaps the % decrease that corresponds to the 645 number could be added so that readers can make a parallel comparison?

Answer: Thank you for noting this. We have exchanged absolute numbers by % consistently throughout the text to make it easier to follow. In this way, reader can better identify the additional effort of each scenario compared to the reference ones.

[Comment #3.11]

The time periods presented in the text do not always match. Figure 2 shows 2020-2050. The methods state that the start year is 2020, but the discussion of China's results make comparisons between 2015 and 2050. A minor related point: the supplement lists GDP in 2005 USD - presumably these are 2020 GDP numbers, why state in 2005 dollars?

Answer: We have updated the draft and all the statements (including GDP numbers). Now, they consistently refer to 2020.

[Comment #3.12]

Is there meant to be a parallel between Figures 1 and 3? Some of the panels are different. There seems to be some overlap with Figure 4 as well – is this supposed to show only relative changes, while the comparison of Figures 1 and 3 shows absolute results? Perhaps there is a clever way to combine some of this information, or also show the RS results in Figure 3. Otherwise, I found myself constantly flipping back to earlier graphs to make comparisons.

Answer: We have provided in the text more clarity on the logics behind the figures, as well as updated Figures 1 and 3. Figure 1 reflects the status quo (year 2020), whereas Figure 3 reflects the situation under the DS in 2050.

[Comment #3.13]

Socio-economic insights section, the 8100 kWh/cap result is duplicated in this paragraph. Also, it looks like there is a typo, it should be 100 m2/cap, not 1000.

Answer: Thank you for noticing, we have updated the text accordingly.

[Comment #3.14]

Figure 4, the connecting lines in the lower-right panel are a nice visual aid – I would recommend including for all of the panels.

Answer: Thanks for this suggestion. We have updated Figure 4 including lines in all panels. To increase the readability, we have also added an oval arrow to indicate the direction of the evolution over time, where the oval indicates the value for 2050.

[Comment #3.15]

It doesn't appear that future climate change is accounted for here – this should be noted somewhere.

Answer: We have added a sentence clarifying that climate change is not included at the end of the first paragraph of section "Status quo: socio-economic, climate and energy indicators".

[Comment #3.16]

I wish the results had more explanatory power. It isn't clear why what is driving the differences in results, and the discussion doesn't take full advantage of the bottom-up modeling framework. It would be good to highlight exactly what knowledge was gained here, as distinct from insights that are possible from integrated assessment modeling. For example, Figure 4 upper-right panel shows building energy demand vs carbon intensity, but it would be much more interesting to see emissions vs carbon intensity. Very few physical descriptors of the actual building stock are presented, really only floorspace. It would be good to see average U-values for example, or penetration of certain technologies.

Answer: This comment has been addressed in a number of ways. First, we have included explanations as to what are driving the various models in the text and why the various modelling results might vary across Europe. We have also replaced the bottom right chart of Figure 4 by average emission per capita vs carbon intensity of energy consumption, which is indeed more coherent with the structure. Furthermore, we have added the U-values for the RS in 2020 in SM1, as well as U-values for DS 2050 in SM2.

[Comment #3.17]

How do these results compare with those derived from national or international energy models? Do the bottom-up results corroborate existing aggregated results, or do they reveal patterns that were not evident previously?

Answer: We have made a number of amendments to the study to address this comment. We have included additional information in the conclusion section to describe this. Furthermore, we have complemented this explanation with a summary data table as a Supplementary Material 5.

[Comment #3.18]

My last comment is the most general: it is hard to get a clear picture of the results because the models and scenarios being employed are different. This appears to be the strategy of the authors and there is no getting around it as each bottom up model was constructed independently. But, putting the results together side-by-side requires more discussion of proper interpretation for readers. The authors have done a nice job summarizing each model in the methods section, giving high-level information in Table 1, and presenting details in the Supplement. I would highly recommend some additional text to discuss common features across models. It should be clarified if all models are covering the same suite of energy end uses and technology options. It should be clarified if all models are considering the same suite technology options. For example, do all models consider appliance efficiency upgrades, or ground-source heat pump conversion, or is the technology suite considered country-dependent? Are new buildings built to different codes in each model, and what is the influence of new construction vs retrofitting existing buildings in each region? And for the DS scenarios, if the actual background assumptions are completely different, how are readers supposed to interpret the results? If Europe decarbonizes its buildings more rapidly than the US and China, is this because the scenarios are not equivalent, more than the effect of population or status of the current building stock? Perhaps more quantitative information about the scenarios can be added to Table 1. In the end, if in fact the models and the scenarios are completely different and there is no fair way to compare results across countries, then the authors should be very careful in their discussions

to caution readers and to explain that the results simply represent the most ambitious scenarios being considered in each country, and that even the formulation of the scenarios is a reflection of what national modelers believe is achievable.

Answer: In order to address this comment, we have now made clear in the beginning of the paper that the suit of measures is country-dependent based on the interventions deemed as viable by each modelling, removing the word “Ambitious” from the definition, so that we now have “Decarbonisations Scenarios (DS)” instead of “Ambitious Decarbonisations Scenarios (DS)”. To ensure that readers are aware that the results simply represent the most ambitious scenarios being considered in each country, and that the formulation of the scenarios is based on a reflection of what national modellers believe is achievable, explanations have been added throughout the storyline, i.e., the initial section, discussion, and method section.

Further, we have inserted a new column in SM2.1 titled “Technology and demand development”, which addresses the energy end uses and technology options covered by each model.

Building codes are country-specific and their key aspects have been defined in SM2, column “Policy instruments”. In parallel, for each region, in order to depict the influence of new construction versus retrofitting existing buildings (along with implications for achieving carbon neutrality), we have included new construction and renovation rates in SM2 column, now re-named as “Demographic and floor area development”. Likewise, more quantitative information about the scenarios has been added to SM2, column “Scenario assumptions”.

Reviewer comments, second round

Reviewer #1 (Remarks to the Author):

I am impressed by how much better the paper has become in light of the revisions. I think it's potentially publishable in Nature Communications now. I am still unsure, as I feel there isn't a key finding or set of findings that go beyond just the reporting of results. I have one further substantive comment, which is that I believe the "Methods" would benefit from a little more discussion of how the scenarios in each region were defined, and why they are seen as realistic decarbonisation scenarios, considering renovation rates, electricity decarbonisation rates, etc.

I have two minor comments:

- please ensure units are complete and accurate, e.g. line 328 should say gCO2/kWh (assuming this is what is meant when describing the carbon intensity of electricity).

- why the focus on RES only in centralised electricity? e.g. Line 326 states that the share of renewable energy sources in the energy (should this be electricity?) mix needs to increase. Shouldn't it be broader, and discuss the role of zero-carbon sources including nuclear?

Reviewer #2 (Remarks to the Author):

The authors revised the paper in detail according to the remarks of each reviewer. There are a few points for the authors to consider to improve the paper:
(1) Abbreviation of all the teams in the manuscript shall be given at first.
(2) "Heating and cooling demand also increases with economic
growth, with cooling loads in China's northern climate zones rising from 5 – 7 kWh/m2 227 in 2020 to 4 –19 kWh/m2 in 2050. In the transition climate zones, heating loads rise from ~25 kWh/m2 228 to 10-68kWh/m2 229 over the same time period." What is the time period of the "cooling loads" and "heating loads"?
(3) Since it is a collaborative work finished by researchers from various countries, it is obvious that different researchers might have different ways of writing and data analysis. However, the chief author shall try to make the entire paper in the same style.

Reviewer #3 (Remarks to the Author):

The authors have provided detailed responses and revisions to reviewer comments, most of which are satisfactory.

I think the most important theme of the comments is about how readers should interpret the novelty of insights provided by the work. The authors claim that their compilation of national models allows for disaggregation and also avoids some unrealistic blanket assumptions that have been used in global models. In the revised text, they point out for example

"Our assumptions for developed economies and conclusions are aligned with observed trends of declining household sizes28-30 but run contrary to the embedded assumptions of maintaining or reducing floor areas per capita in many global models 32,33 297 (see Supplementary Material 5)."

I would like to see more insights like this. Otherwise, Supplementary Table 5 is not really highlighted elsewhere in the Methods or Discussion sections. I would recommend a stronger explanation in the main text about the advantages of this national model compilation approach that the authors have laid out in the response to reviewers document, specifically highlighting disaggregated results that are not available from the global models.

There is some general text in the Conclusion:

“Renovation rates (1.4%), onsite RES (38%), electrification of buildings total FEC (38-80%), and demand reductions driven by lifestyle changes, including small changes in everyday practices as well as densification of building occupancy, appear to be overlooked in the considered DS of the national building sector models, but are key drivers of the transition of the building sector according to decarbonisation pathways identified in global models.”

I don’t really understand this, perhaps the authors can add some clarification. The implication is that these factors are included in global models but are not included in the current manuscript. If that is so, from where are these values derived? In the rebuttal letter, the authors stated that their models are avoiding some unrealistic global modeling assumptions – is the implication that these factors are not realistic and shouldn’t be included? The word ‘overlooked’ suggests some sort of error, but some factors like renovation rates are clearly included.

The authors go on in the Conclusion to critique the national decarbonization scenarios, stating that they need to be made more ambitious in order to meet climate goals. But tuning the model scenario to meet a goal is not really the objective, it might be better to say that the national decarbonization plans that the scenarios are meant to represent need to be made more ambitious.

REVIEWER COMMENTS

Reviewer #1 (Remarks to the Author):

I am impressed by how much better the paper has become in light of the revisions. I think it's potentially publishable in Nature Communications now. I am still unsure, as I feel there isn't a key finding or set of findings that go beyond just the reporting of results.

Answer: Thank you very much for the positive feedback as well as for the revision points shared in the previous and this revision that has helped us substantially improve the paper.

The key finding of the study is the demonstration that DS can lead to substantial carbon reductions within the range projected in the 2°C degrees scenario but still insufficient to achieve the 1.5°C degrees scenario. Such a detailed analysis —considering techno-economic and policy feasibility within each national context— had not been done before using building stock bottom-up building sectoral models covering so many world regions. This work is a pioneer attempt to validate results from global models using bottom-up models, which are more detailed and include specificities related to the building stock, energy markets, user preferences and policies.

Global models typically do not represent the demand in the building stock — but rather the economy and the energy system as a whole — and therefore cannot estimate what are the viable constructive solutions and technologies that can be implemented in each context to reduce energy demand and derived energy-related carbon emissions. In our study, modelers for each country have an accurate understanding on the building stock, potential retrofit measures, building construction possibilities as well as associated energy demand and carbon emissions in each case. To date and to the best of our knowledge, this is the most accurate, granular and at the same time broad (i.e., number of countries and regions) analysis of carbon mitigation scenarios in buildings that has ever been developed. Such an analysis – and respective comparison and discussion with results from global models – was missing. This helps to inform each of the models and represents what we believe is a solid contribution to our field as it reinforces global assessments to estimate the achievement of climate goals by the building sector.

To highlight this message in the manuscript, we have adapted the introduction (lines 61 and 67), methodology (lines 381 to 389), results (lines 266 to 274), and conclusions sections (lines 253 to 358 and 374 to 379).

I have one further substantive comment, which is that I believe the "Methods" would benefit from a little more discussion of how the scenarios in each region were defined, and why they are seen as realistic decarbonisation scenarios, considering renovation rates, electricity decarbonisation rates, etc.

Answer: We have updated the methods section to describe further bottom-up building sectoral models. Given the word limitation, we were not able to present in the main text the full details of how the scenarios in each region were defined. Instead, in the introduction section we inform the reader that the discussion of the main drivers behind technology and demand developments (i.e., policy instruments, energy price, demographic and floor area, demand drivers, primary model inputs, etc.), for each of the models and scenarios can be

accessed in the Supplementary Material 2 (see lines 92 to 96).

I have two minor comments:

- please ensure units are complete and accurate, e.g. line 328 should say gCO2/kWh (assuming this is what is meant when describing the carbon intensity of electricity).

Answer: We have updated the units accordingly, thanks for noticing.

- why the focus on RES only in centralised electricity? e.g. Line 326 states that the share of renewable energy sources in the energy (should this be electricity?) mix needs to increase. Shouldn't it be broader, and discuss the role of zero-carbon sources including nuclear?

Answer: We have now improved the text to make clear that we do not limit the deployment of electricity from RES, depending on the national context, different assumptions are taken on the energy sources beyond electricity, such as bioenergy (e.g. NW Europe in the Invert/EE-Lab). However, it is clear that electrification is pivotal when it comes to decarbonising the building sector due to the fact that RES electricity — wind and solar — will continue to grow in the coming years. Then, we do not exclude any RES but electricity-based RES has a major role.

Regarding nuclear, the different models have tackled it indirectly. Whereas for some models it is explicit in the fuel mix of the energy system, in other models (Invert/EE-Lab, CoreBee or ECCABS), it has been accounted for indirectly through the assumed changes in the carbon intensity of electricity production.

We have also explained in the commentary of SM5, that in line 151 we focus on the share of electricity from RES, because it is a key indicator for which explicit information is provided in all the studies included in the comparison presented in SM5.

Reviewer #2 (Remarks to the Author):

The authors revised the paper in detail according to the remarks of each reviewer. There are a few points for the authors to consider to improve the paper:
(1) Abbreviation of all the teams in the manuscript shall be given at first.

Answer: We appreciate the reviewer´s concern and have carefully checked the journal guidelines for submission and many of the journal recent papers (of similar topics and published over the past years) and find no requirements to compile in a table in the beginning of the article the abbreviations of the key terms used in the manuscript. Given the word cap, and that the abbreviations we use are introduced the first time they are used in every case, we hope that the reviewer could accept to leave it up to the editorial team to decide if such a table is necessary.

(2) "Heating and cooling demand also increases with economic growth, with cooling loads in China’s northern climate zones rising from 5–7 kWh/m2 227 in 2020 to 4–19 kWh/m2 in 2050. In the transition climate zones, heating loads rise from ~25 kWh/m2

228 to 10-68kWh/m2 229 over the same time period." What is the time period of the "cooling loads" and "heating loads"?

Answer: We have modified the text to make it clear that we refer to energy (kWh) and not to loads (kW). In other words, we provide values for the heating and cooling energy needs over a typical year. Specifically, in line 252 and 254, we have replaced the terms 'cooling load' and 'heating load' by 'cooling demand per unit floor area' and 'heating demand per unit floor area' respectively.

In addition to the above modification, we would like to clarify that we provide ranges because we have different commercial building types (office, retail, hospital, hotel, school etc.). For the case of China, office buildings could be the most representative among all commercial building types. We also keep different vintages of buildings, such as new, ultra-low new, existing, retrofitted existing, deep retrofit and so on. Each of the vintage types has different cooling/heating demands. Such ranges are however not calculated in all the modelling exercised compiled in this work. This depends on how each model calculates the energy demand for the building sector. To make this clear in the manuscript we have included the following footnote (**) for the column 'Spatiotemporal resolution' in table 1: ** *Each model makes specific assumptions for the calculation of the energy requirements. This includes decisions on the level of resolution, geographical scope or set-point temperatures among others.*

(3) Since it is a collaborative work finished by researchers from various countries, it is obvious that different researchers might have different ways of writing and data analysis. However, the chief author shall try to make the entire paper in the same style.

Answer: Indeed, to harmonize the style, the manuscript has been revised by an external professional language proof service before this last submission. You may therefore observe comprehensive editorial changes through the entire paper to harmonize the style and remove any typos.

We have also attached a certificate from the proof-reading service verifying that the article has been professionally proofread with special attention to meeting the Journal's author guidelines for formatting, structure and referencing.

Reviewer #3 (Remarks to the Author):

The authors have provided detailed responses and revisions to reviewer comments, most of which are satisfactory.

I think the most important theme of the comments is about how readers should interpret the novelty of insights provided by the work. The authors claim that their compilation of national models allows for disaggregation and also avoids some unrealistic blanket assumptions that have been used in global models. In the revised text, they point out for example

Answer: We have now stressed the contribution of the bottom-up building sectoral models in the following sections; results (lines 266 to 274), conclusions (lines 352 to 358 and 374 to 279) and methodology (lines 381 to 389). We have also updated the title to better reflect the aim of the paper. Please see our reply to the first comment by Reviewer #1 above, which was

very much aligned with this comment of Reviewer #3.

“Our assumptions for developed economies and conclusions are aligned with observed trends of declining household sizes28-30 but run contrary to the embedded assumptions of maintaining or reducing floor areas per capita in many global models 32,33 297 (see Supplementary Material 5).”

I would like to see more insights like this. Otherwise, Supplementary Table 5 is not really highlighted elsewhere in the Methods or Discussion sections. I would recommend a stronger explanation in the main text about the advantages of this national model compilation approach that the authors have laid out in the response to reviewers document, specifically highlighting disaggregated results that are not available from the global models.

Answer: As the reviewer points out, we are now providing the full comparison in SM5. We have also included additional clarifications in the conclusion and method sections. We have also improved the text (and SM5) with comments on the difference between 1.5°C–2°C targets, which based on your review we realized is a key finding of our work that was not sufficiently stressed in the previous draft. We find, however, challenging to provide full details in the main text due to word-limit, and have addressed the readers to the Supplementary Materials and references therein for the most comprehensive information, which we have completed carefully.

There is some general text in the Conclusion:
“Renovation rates (1.4%), onsite RES (38%), electrification of buildings total FEC (38-80%), and demand reductions driven by lifestyle changes, including small changes in everyday practices as well as densification of building occupancy, appear to be overlooked in the considered DS of the national building sector models, but are key drivers of the transition of the building sector according to decarbonisation pathways identified in global models.”

I don’t really understand this, perhaps the authors can add some clarification. The implication is that these factors are included in global models but are not included in the current manuscript. If that is so, from where are these values derived? In the rebuttal letter, the authors stated that their models are avoiding some unrealistic global modeling assumptions – is the implication that these factors are not realistic and shouldn’t be included? The word ‘overlooked’ suggests some sort of error, but some factors like renovation rates are clearly included.

Answer: In this paragraph we present a comparison of implementation rates of mitigation measures assumed in this study to the rates of similar measures included in the global models (SM5). The currently assumed rates in our study for: renovation (1.4%), share of RESs (38%), and share of electricity (38%–80%) may not be sufficient to reach the temperature goal of 1.5°C. Therefore, beyond the efforts implied in the sectoral models, decarbonization scenarios in line with 1.5°C target would require additional efforts deriving in at least 1% higher renovation rate, 4%–14% larger share of electricity, and 3%–30% larger share of renewables. Likewise, the carbon intensity of electricity production should be 3–7 times less than that assumed in the DSs considered in this study.

We have rephrased the paragraph in the conclusions section (lines 350 to 379) and in SM5 (lines 28 to 89) to ensure message that the message is now clear.

The authors go on in the Conclusion to critique the national decarbonization scenarios, stating that they need to be made more ambitious in order to meet climate goals. But tuning the model scenario to meet a goal is not really the objective, it might be better to say that the national decarbonization plans that the scenarios are meant to represent need to be made more ambitious.

Answer: We really appreciate this observation. We have revised the conclusion section to ensure that the message is clear now (lines 367 to 373); we conclude that DS scenarios can achieve significant CO2 emissions reductions but still insufficient to achieve the ambitions set by the 1.5-2 °C scenarios. From these results we don't imply that national models should be redesigned to reach the 1. 5-2 °C. Instead, we discussed — as we indicated in an earlier revision point — which are the reason behind them (i.e., assumption on the carbon intensity factor for electricity by 2050. Along the same lines, we cannot conclude that global scenarios are not reasonable nor feasible. We compare both (bottom-up and global models) and discuss their differences having in mind that sectoral models provide better level of detail and that the design of global scenarios should reflect upon results from those models.

** See Nature Research's author and referees' website at www.nature.com/authors for information about policies, services and author benefits.

This email has been sent through the Springer Nature Tracking System NY-610A-NPG&MTS

Confidentiality Statement:

This e-mail is confidential and subject to copyright. Any unauthorised use or disclosure of its contents is prohibited. If you have received this email in error please notify our Manuscript Tracking System Helpdesk team at http://platformsupport.nature.com .

Details of the confidentiality and pre-publicity policy may be found here http://www.nature.com/authors/policies/confidentiality.html

Privacy Policy | Update Profile

Reviewer comments, third round

Reviewer #1 (Remarks to the Author):

Thanks for these further revisions. I am happy with the paper now and happy for it to be published.

Reviewer #2 (Remarks to the Author):

The paper has been improved a lot by the authors according to the reviews' remarks. I think it is ready for publishing.

Reviewer #3 (Remarks to the Author):

I looked through all of the authors' responses to reviewer comments and revisions and found them all to be satisfactory. I think there is now enough interpretation that readers can get a fair sense of the ensemble message with these disparate models.
