# Supplementary material for: A global comparison of building decarbonization scenarios by 2050 towards 1.5–2 °C targets
Source: Nat Commun. 2022 Jun 2;13:3077. doi: 10.1038/s41467-022-29890-5 (PMC9163154; doi:10.1038/s41467-022-29890-5)
Supplement: Supplementary file 2 — Peer Review File [file 41467_2022_29890_MOESM2_ESM.docx]

| **Author Checklist** | NCOMMS-21-03413B |
| --- | --- |
| **0000000000000000000000000000000000000000000000000000000000000** | **0000000000000000000000000000000000000** |
| **Please check the items below carefully and add a response in each row of the table to indicate the changes that you have made. Please also check through any additional marked-up edits we may have provided within the manuscript file.** | |
|  |  |
|  |  |
|  |  |
| Abstract and editor's summary |  |
| Our guidance: | Your response: |
| Please edit the title so that it is 15 words or fewer and does not include punctuation. | Done |
| Please remove the final sentence of the Abstract. | Done |
|  |  |
| Author information |  |
| Our guidance: | Your response: |
|  |  |
| Please review your complete author list to verify that it is complete and accurate. We ask that you consult with your coauthors to ensure that all names, affiliations, and titles are represented correctly. Note that if any authors are added or removed after this point then all authors will be requested to provide approval documentation that could potentially delay the production of your paper. | Done |
| Please include a list of the corresponding authors and an e-mail address for each one in the final version of the manuscript file. | Done |
| Please ensure that all affiliations are in the correct sequential order according to their position in the author list. Affiliation 1 must be the first affiliation associated with the first author. Please see this article as an example: https://www.nature.com/articles/s41467-020-16621-x.pdf | Done |
|  |  |
| Article structure |  |
| Our guidance: | Your response: |
| We can accommodate up to 10 display items (Figures or Tables) in the main article. Each Figure and Table must fit easily within an A4 page (210 x 297 mm). Please ensure that the number and size of your Figures and Tables fulfil these requirements to avoid any delay in the acceptance of your article. | Done |
| **Please ensure your main manuscript file includes the following sections, in this order:** |  |
| *Title Author list Affiliations Abstract Introduction Results Discussion (optional) Results and Discussion (optional) Methods (including Data Availability, Code Availability and Statistics subsections where relevant) References Acknowledgements Author Contributions Statement Competing Interests Statement Tables Figure Legends/Captions (for main text figures)* | *Done* |
| We do not edit Supplementary Information files; they will be uploaded with the published article as they are submitted with the final version of your manuscript. Any tracked changes should be removed from the file and the file should be provided as a PDF file. Supplementary Figures do not need to be provided separately. |  |
|  |  |
|  |  |
| Main text |  |
| Our guidance: | Your response: |
| Please rearrange the Introduction so that all discussion of previous work appears first. The final paragraph should contain only a concise summary of the current work, in the present tense, and begin with a phrase like “In this work” or “Here, we show”. | Done (lines 102-109) |
| Please divide the Results section into subsections, each with a title of 60 characters or fewer including spaces. | Done |
| Please remove or rename the Conclusion heading, as the main text should only include the sections Abstract, Introduction, Results, optional Discussion and optional Methods. We also allow a combined Results and Discussion section. | Done, We have replaced “Conclusions” by “Discussion” |
| Please do not use italics, bold font, underlining or speech marks unless required for technical terms (in both the main text and the display items). | Done. We only have italics in the name of the models and scenarios, as requested in the “Guide to formatting articles”. |
| Please make sure that mathematical terms throughout your manuscript and Supplementary Information (including in figures, figure axes, and legends) conform strictly to the following guidelines. Equations must be supplied in editable format, and not as images. Scalar variables (e.g. x, V, χ) must be typeset in italic, whereas multi-letter variables and functions (e.g. log) must be formatted in roman. Vectors (such as the wavevector k or the magnetic field vector B) must be typeset in bold without italics. | Done |
| The results of this study should only be discussed in the final paragraph of the Introduction. This paragraph should provide a short summary of the main findings of your study. Technical details that are necessary to follow the main text should be provided at the beginning of the Results section, while a more detailed discussion should be placed in the Methods section at the end of the article. Please focus on what you find, not what you do in the introduction. | Done |
| Please remove punctuation (colons etc.) from section headings/subheadings where possible. | Done |
|  |  |
| Figures and Tables |  |
| Our guidance: | Your response: |
| Please see the guidelines linked below for detailed instructions about how your figures should be prepared. Following these instructions will reduce the chances of delays should we need to request replacement artwork from you at a later stage. | Done |
| <https://www.nature.com/documents/NRJs-guide-to-preparing-final-artwork.pdf> |  |
| If bold/italic formatting in tables is necessary, please define its meaning in a table footnote | Done |
| Tables may not contain dividing lines within cells - please reformat | Done |
| Shadings or symbols in graphs must be defined in some fashion. We prefer that you use a key within the image; do not include colored symbols in the legend/caption. | Done |
| Every figure panel must be described in the legend/caption. | Done |
| Each display item should be no larger than a single A4 page (260x179 mm). | Done |
| Any abbreviations, symbols or colours present in your figures must be defined in the associated legends. | Done |
| We recommend moving Fig. 1 in the Introduction section to the Methods or Results section. | We have tried to move Fig. 1 to the Methods section but then it was impossible to follow the storyline. We would highly suggest to keep it where it is. |
| Please note that we do not allow for footnotes/notes in Figures. | Done |
|  |  |
| Data and Code |  |
| Our guidance: | Your response: |
| Nature journals strongly support public availability of data and code. Please deposit the data and code used in your paper into a public data repository, or alternatively, present the data as Supplementary Information. If data can only be shared on request, please explain why in your Data Availability Statement, and also in the correspondence with your editor.   Please note that for some data types, deposition in a public repository is mandatory. Any restrictions on sharing of these data types must be clearly indicated in the statement and discussed with the editor. More information on our data deposition policies and available repositories can be found here: | Done |
| <https://www.nature.com/nature-research/editorial-policies/reporting-standards#availability-of-data> |  |
| All published manuscripts reporting original research in Nature Portfolio journals must include a data availability statement, as a separate section before the References and under the heading 'Data Availability'.   The data availability statement must make the conditions of access to the “minimum dataset” that are necessary to interpret, verify and extend the research in the article, transparent to readers.   This minimum dataset may be provided through deposition in public community/discipline-specific repositories, custom proprietary repositories or general repositories like Figshare, Zenodo and Dryad. Providing large datasets in supplementary information is strongly discouraged and the preferred approach is to make data available in repositories. Scientific Data, a Nature Portfolio journal, maintains a list of approved and recommended data repositories to support researchers seeking suitable repositories for their data (https://www.nature.com/sdata/policies/repositories).  The Data Availability Statement should also reference any source data published alongside the paper.  If DOIs are provided, we also strongly encourage including these in the Reference list (authors, title, publisher (repository name), identifier, year).  For clinical datasets or third party data, please ensure that the statement adheres to our policy (https://www.nature.com/nature-research/editorial-policies/reporting-standards#availability-of-data) | Done |
| Please use the following template to provide all the information stated above:  The XX data generated in this study have been deposited in the YY database under accession code ZZ [add hyperlink here]. The XX data are available under restricted access for {insert reason}, access can be obtained by {explain how}. The raw XX data are protected and are not available due to data privacy laws. The processed XX data are available at YY. The XX data generated in this study are provided in the Supplementary Information/Source Data file. The XX data used in this study are available in the YY database under accession code ZZ [Add hyperlink here]. | Done |
|  |  |
| Methods |  |
| Our guidance: | Your response: |
| Sufficient details of the experiments must be provided in the Methods section such that they could be reproduced without reference to published papers. Use of the term "as described previously" is not encouraged. | Done |
|  |  |
| References |  |
| Our guidance: | Your response: |
| All references must be cited in numerical order. The reference list will be formatted according to the Nature style by our journal production team, however please ensure that references contain all of the information required, eg:  Kurumada, S., Takamori, S. & Yamashita, M. An alkyl-substituted aluminium anion with strong basicity and nucleophilicity. <i>Nat. Chem.</i> <b>12</b>, 36–39 (2020). | Done |
|  |  |
| End matter |  |
| Our guidance: | Your response: |
| Nature Portfolio defines Competing Interest (CI) as financial and non-financial interests (including but not limited to funding, employment, stocks, shares, patents, personal or professional relationships with individuals or institutions, and unpaid membership advocacy) that could be perceived to directly undermine the objectivity, integrity, and value of a publication, or could be seen as having an influence on the judgments and actions of authors with regard to objective data presentation, analysis, and interpretation.  Please thoroughly review our policy on Competing Interests and include a detailed statement both in your final manuscript file and in our manuscript tracking system. Please ensure the statements are identical in both. Be specific about how each point stated relates to the research and list applicable author initials, and/or patent numbers.  If there are no competing interests, a negative statement must be included. |  |
| <https://www.nature.com/nature-research/editorial-policies/competing-interests> |  |
| Please confirm that all relevant funding awarded to each author is described in the Acknowledgements section. List each grant number, followed by the initials of the author who received it. | Done |
|  |  |
| Preparing your manuscript files |  |
| Our guidance: | Your response: |
| Unless otherwise stated please limit individual file sizes to approximately 30MB. We strongly encourage the use of repositories for large datasets or source data due to size considerations. | Done |
| Please supply a brief (maximum 250 characters, including spaces) summary of the main findings of the paper to be used on our website and in our e-alerts. The summary should be written in the third person in language suitable for a broad audience. The summary may be edited by the editors prior to publication. Please provide this summary in your cover letter. | Done |
| To ensure maximum visibility for your work, we may tweet about your paper following publication. If you would like us to include the Twitter handles of the first author(s), corresponding author(s), lab or institution in this tweet, please provide them in your cover letter. We would also welcome your suggestions for hashtags to use when tweeting about the work. | @jpjimeneznav @Clara_Camarasa @filippidoufaid1  @HolckNina @Erikamata_tw #climatechange  #energytwitter  #buildings #netzerocarbon |
| Please provide figures as individual vector files with editable text. Acceptable file types for figures are .ai, .eps, .pdf or Chem Draw for fully editable vector-based art. For detailed guidance on figure preparation, see https://www.nature.com/documents/aj-artworkguidelines.pdf | Done (Figure X.pdf, as indicated in the Cover Letter) |
| Please supply the main Supplementary Information file as a single PDF file. | Done |
| All Supplementary Information files (e.g. Supplementary Data, Supplementary Software, etc.) must be cited in the main text. | Done |
| All Supplementary Information items (e.g. Supplementary Figures, Supplementary Tables, Supplementary Methods, Supplementary Notes, Supplementary Discussion, Supplementary References) must be included in one PDF document. Only Supplementary Movie, Audio, Data and Software files should be submitted separately from the Supplementary Information. | Done |
| The use or adaptation of previously published images is strongly discouraged. If this is unavoidable, please request the necessary rights documentation to re-use such material from the relevant copyright holders and return this to us when you submit your revised manuscript. Please check whether your manuscript or Supplementary Information contain third-party images, such as figures from the literature, stock photos, clip art or commercial satellite and map data.  For more information on what constitutes ownership by a third party, please contact our Editorial Assistant at naturecommunications@nature.com | Done, we don’t use previously published images. |
|  |  |
|  |  |
| Forms to complete |  |
| Our guidance: | Your response: |
| **Editorial Policy Checklist** |  |
| Please update and upload a final version of the Editorial Policy Checklist with your revised manuscript files. A blank Editorial Policy Checklist can be found via the link below. Note that this form is a dynamic ‘smart pdf’ and must be downloaded and completed in Adobe Reader. |  |
| Please update your current checklist or download from: |  |
| <https://www.nature.com/documents/nr-editorial-policy-checklist.zip> | Done |
|  |  |
|  |  |
| **You will need to upload:** |  |
| Editorial Policy Checklist | Done |
| Completed Third Party Rights Table (if relevant) | Not relevant |
| A point-by-point response to the reviewers' comments | No comments pending from reviewers |
| A completed copy of this checklist | Done |
| The main article file in Microsoft Word format - please supply a version with tracked changes and a version with tracked changes accepted | Done |
| Separate Figure files | Done (in Excel and in PDF) |
| Inventory of Supporting Information | Done, also included in the Cover Letter |
| A Supplementary Information file | Done |
| Reporting Summary | We don’t know what is referred to |
